# Recommendations for quantifying and reducing uncertainty in climate projections of species distributions

**DOI:** 10.1111/gcb.16371

**Published:** 2022-08-17

**Authors:** Stephanie Brodie, James A. Smith, Barbara A. Muhling, Lewis A. K. Barnett, Gemma Carroll, Paul Fiedler, Steven J. Bograd, Elliott L. Hazen, Michael G. Jacox, Kelly S. Andrews, Cheryl L. Barnes, Lisa G. Crozier, Jerome Fiechter, Alexa Fredston, Melissa A. Haltuch, Chris J. Harvey, Elizabeth Holmes, Melissa A. Karp, Owen R. Liu, Michael J. Malick, Mercedes Pozo Buil, Kate Richerson, Christopher N. Rooper, Jameal Samhouri, Rachel Seary, Rebecca L. Selden, Andrew R. Thompson, Desiree Tommasi, Eric J. Ward, Isaac C. Kaplan

**Affiliations:** ^1^ Institute of Marine Sciences University of California Santa Cruz Monterey California USA; ^2^ Environmental Research Division, Southwest Fisheries Science Center, National Marine Fisheries Service National Oceanic and Atmospheric Administration Monterey California USA; ^3^ Southwest Fisheries Science Center, National Marine Fisheries Service National Oceanic and Atmospheric Administration San Diego California USA; ^4^ Alaska Fisheries Science Center, National Marine Fisheries Service National Oceanic and Atmospheric Administration Seattle Washington USA; ^5^ Environmental Defense Fund Seattle Washington USA; ^6^ Physical Sciences Laboratory, Earth System Research Laboratories National Oceanic and Atmospheric Administration Boulder Colorado USA; ^7^ Northwest Fisheries Science Center, National Marine Fisheries Service National Oceanic and Atmospheric Administration Seattle Washington USA; ^8^ Cooperative Institute for Climate, Ocean, and Ecosystem Studies University of Washington Seattle Washington USA; ^9^ Ocean Sciences Department University of California Santa Cruz Santa Cruz California USA; ^10^ Department of Ecology, Evolution, and Natural Resources Rutgers University New Brunswick New Jersey USA; ^11^ ECS Tech, in support of, NOAA Fisheries Office of Science and Technology Silver Spring Maryland USA; ^12^ Pacific Biological Station Fisheries and Oceans Canada Nanaimo British Columbia Canada; ^13^ Department of Biological Sciences Wellesley College Wellesley Massachusetts USA

**Keywords:** artificial intelligence, climate change, earth system models, extrapolation, fisheries, machine learning, species distribution models, virtual species

## Abstract

Projecting the future distributions of commercially and ecologically important species has become a critical approach for ecosystem managers to strategically anticipate change, but large uncertainties in projections limit climate adaptation planning. Although distribution projections are primarily used to understand the scope of potential change—rather than accurately predict specific outcomes—it is nonetheless essential to understand where and why projections can give implausible results and to identify which processes contribute to uncertainty. Here, we use a series of simulated species distributions, an ensemble of 252 species distribution models, and an ensemble of three regional ocean climate projections, to isolate the influences of uncertainty from earth system model spread and from ecological modeling. The simulations encompass marine species with different functional traits and ecological preferences to more broadly address resource manager and fishery stakeholder needs, and provide a simulated true state with which to evaluate projections. We present our results relative to the degree of environmental extrapolation from historical conditions, which helps facilitate interpretation by ecological modelers working in diverse systems. We found uncertainty associated with species distribution models can exceed uncertainty generated from diverging earth system models (up to 70% of total uncertainty by 2100), and that this result was consistent across species traits. Species distribution model uncertainty increased through time and was primarily related to the degree to which models extrapolated into novel environmental conditions but moderated by how well models captured the underlying dynamics driving species distributions. The predictive power of simulated species distribution models remained relatively high in the first 30 years of projections, in alignment with the time period in which stakeholders make strategic decisions based on climate information. By understanding sources of uncertainty, and how they change at different forecast horizons, we provide recommendations for projecting species distribution models under global climate change.

## Graphical abstract

Projecting the future distributions of species has become critical for climate adaption planning, but we don't have a precise understanding of how accurate projections can be without waiting decades for validation. Here, we model simulated species responses under climate change to quantify and understand sources of uncertainty, and how they change at different forecast horizons. We provide recommendations for projecting species distribution models under global climate change.

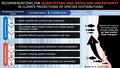

## 1 INTRODUCTION

Climate variability and change is already drastically altering the structure and function of ecosystems globally (Scheffers et al., 2016; Walther et al., 2002). Many species have shifted their distributions in response to climate‐driven changes in the environment, resulting in the largest redistribution of biodiversity since the Last Glacial Maximum (Lenoir et al., 2020; Pecl et al., 2017). These shifts are occurring more rapidly in marine ecosystems compared to terrestrial domains (Lenoir et al., 2020; Pinsky et al., 2019), threatening critical habitat for many species, limiting services for millions of people, and introducing new challenges for ocean governance (Pinsky et al., 2018). With global fisheries revenues projected to decline by 7%–10% over the next three decades (Lam et al., 2016), there is an urgent need for resource managers, fisheries, and communities to anticipate and prepare for alternative future ecosystem states (IPCC, 2021).

Predicting when and where species will move is critical to supporting flexible management frameworks that are capable of responding to climate‐driven change (Pinsky et al., 2018; Tommasi et al., 2021). Developing a clear understanding of the range of potential future conditions can help ocean stakeholders prioritize management strategies (Holsman et al., 2020). For example, species distribution models (SDMs) have become a common tool for resource managers to describe and predict species distributions as a function of various biotic and abiotic factors, thereby providing critical insight into core habitats, range shifts, habitat connectivity, and potential impacts of anthropogenic pressures (Elith & Leathwick, 2009; Guisan & Thuiller, 2005; Robinson et al., 2017). SDMs have been used extensively for predicting and projecting changes into the future (Robinson et al., 2017), but not all models perform well when applied to novel conditions (Barnes et al., 2022; Muhling et al., 2020), which raises concerns about the realism of SDM projections. This is particularly pertinent as novel climate conditions emerge (Smith et al., 2022) because SDMs trained on historical conditions typically have less skill at predicting into novel environmental conditions (Muhling et al., 2020). While the goal of long‐term projections is to quantify broad trends and the scope of potential change over time frames long enough for the externally forced climate change signal to emerge (e.g., 30‐year time slices) (Drenkard et al., 2021) rather than predict actual distributions at fine spatial and temporal scales, it is still important to understand where, when, and why projections may become inaccurate or misleading. We address this need by quantifying how uncertainty can propagate through a species distribution projection framework.

Uncertainty in species distribution projections may come from a number of sources (Reum et al., 2020; Thuiller et al., 2019; Tittensor et al., 2021). These include uncertainty associated with different earth system models, from different future scenarios of forcing variables—typically the Representative Concentration Pathway (RCP) emission scenarios (often called “scenario uncertainty”), and internal variability (Cheung et al., 2016; Morley et al., 2020). Similarly, ecological model uncertainty can arise from differences in model type, design, and parameterization. For instance, ecological model uncertainty can stem from differences in the kinds of ecological processes being estimated, such as modeling “fundamental” and not “realized” niches (i.e., not accounting for the additional constraints on spatial distributions coming from population dynamics, resource availability, and species interactions). Uncertainty can also arise from imperfect sampling of the ecosystem, which can introduce bias and inadequately capture the full environmental niche of a species (also called “observation uncertainty”) (Beaumont et al., 2008; Reum et al., 2020). Uncertainty among climate projections is typically characterized by examining model and scenario uncertainty and internal variability (i.e., uncertainty across earth system models or RCP scenarios, or within multiple realizations of the same model and scenario), whereas characterizing uncertainty across projected ecological SDMs has only gained recent attention (e.g., Morley et al., 2020; Santini et al., 2021; Thuiller et al., 2019; Tittensor et al., 2021). There remains a need for a more systematic approach to better understand how different sources of uncertainty from earth system models and ecological models influence the precision of species distribution projections to assess where resources should be focused to reduce uncertainty in such projections, and also to guide their use in planning and decision‐making.

There are several ongoing initiatives on the U.S. West Coast to evaluate how climate change might affect the future of a wide array of commercial and recreational fisheries, and to develop action plans in response to anticipated change (Busch et al., 2016; Crozier et al., 2019; Link et al., 2015). Understanding how climate change will affect the distribution of fish stocks and their availability to fishing communities is critical to planning for a range of contingencies. Three example species archetypes that are important to U.S. West Coast fisheries and resource managers include coastal pelagic species (CPS; e.g., Pacific sardine *Sardinops sagax caerulea*, northern anchovy *Engraulis mordax*), groundfish species (GFS; e.g., Pacific hake *Merluccius productus*, sablefish *Anoplopoma fimbria*), and highly migratory species (HMS; e.g., albacore tuna *Thunnus alalunga*, swordfish *Xiphias gladius*). Different species are often modeled using different approaches by different modeling communities, which can make interspecies and intermodel comparison difficult and hinder our ability to move toward a unified understanding of how species occurrence and fisheries biomass availability are likely to shift under climate change.

There is a large body of literature on projecting species distributions and abundance under climate change, but comparatively few simulation studies (but see Santini et al., 2021). Thus, we do not have a precise understanding of how accurate or uncertain projections can be without waiting decades for validation. Simulations, however, enable a systematic evaluation of SDM performance over climate timescales. We simulate species distributions from 2011 to 2100 to evaluate how well a suite of estimation models (SDMs) captures the true state of the simulated system. Based on predefined relationships with environmental covariates, our simulations of virtual species provide a known truth for validation and experimental testing and provide a framework for developing best practice principles (e.g., Meynard et al., 2019; Zurell et al., 2016). We use the California Current System (CCS) and CPS, GFS, and HMS archetypes for the U.S. West Coast to parameterize our simulations, an approach that is consistent with the need to manage diverse fisheries and species in complex, rapidly changing ocean ecosystems. Importantly, we use regionally downscaled earth system models (ESM) for the CCS to represent relatively fine‐scale environmental variability and important subsurface processes. We designed our simulation experiment to answer four questions:

- How does the use of environmental and spatiotemporal covariates influence SDM projection accuracy?
- How does SDM performance degrade over the projection period?
- How do SDMs perform when predicting to novel environmental conditions?
- What are the dominant sources of uncertainty and how do they change over the projection period?

## 2 METHODS

### 2.1 Summary

**FIGURE 1:**
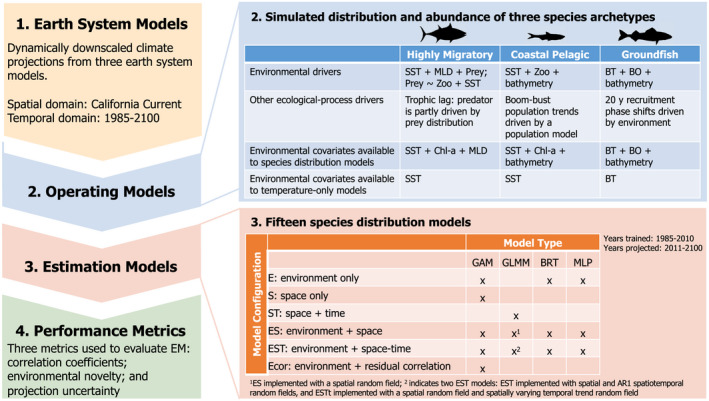
Stepwise conceptual outline of the modeling approach. In step 1, three downscaled earth system models are used as environmental forcing. In step 2, operating models are created for three species archetypes, with a breakout table indicating the ecological and environmental drivers used for each species. In step 3, 15 species distribution models are built for each species archetype, with environmental covariates corresponding to those in step 2. In step 4, three performance metrics are used to compare models (*n* = 252 models) and answer our four study questions. Acronyms in step 2 correspond to sea surface temperature (SST), mixed layer depth (MLD), zooplankton (zoo), bottom temperature (BT), and chlorophyll *a* (Chl‐*a*); and in step 3 correspond to generalized additive model (GAM), generalized linear mixed model (GLMM), boosted regression tree (BRT), and multilayer perceptron (MLP).

We used a combination of regional ocean climate projections and simulated species distributions (Leroy et al., 2016) to quantify sources of uncertainty in projections of spatially explicit biomass for three species archetypes in the CCS (1985–2100; Figure 1). Species archetypes were simplified representations of three general groups of marine finfish found in the CCS that comprise ecologically and/or economically important fisheries and that might be expected to show variable patterns of redistribution under climate change based on their habitat preferences, population dynamics, and mobility characteristics: (1) a highly migratory species (HMS) that was designed to resemble north Pacific albacore; (2) a coastal pelagic species (CPS) that was designed to resemble northern anchovy (CPS); and (3) a groundfish species (GFS) that was designed to resemble sablefish. SDMs (*n* = 15; Figure 1) were then fitted to simulated biomass data for each archetype (training period 1985–2010) and projected from 2011 to 2100 using each of the three regional ocean climate models. Our framework resulted in 252 SDMs (15 SDM types, three species archetypes, three ESMs, and two environmental parameter simulations; Figure 1). To address our study goal of assessing SDM performance and understanding sources of uncertainty in species distribution projections, we compared the output of SDM projections against simulated “observations” for 2011–2100 and quantified the uncertainty introduced by the climate projection (ESM uncertainty) versus the uncertainty introduced by the SDM structure (SDM uncertainty).

### 2.2 Environmental covariates from regional ocean projections

**FIGURE 2:**
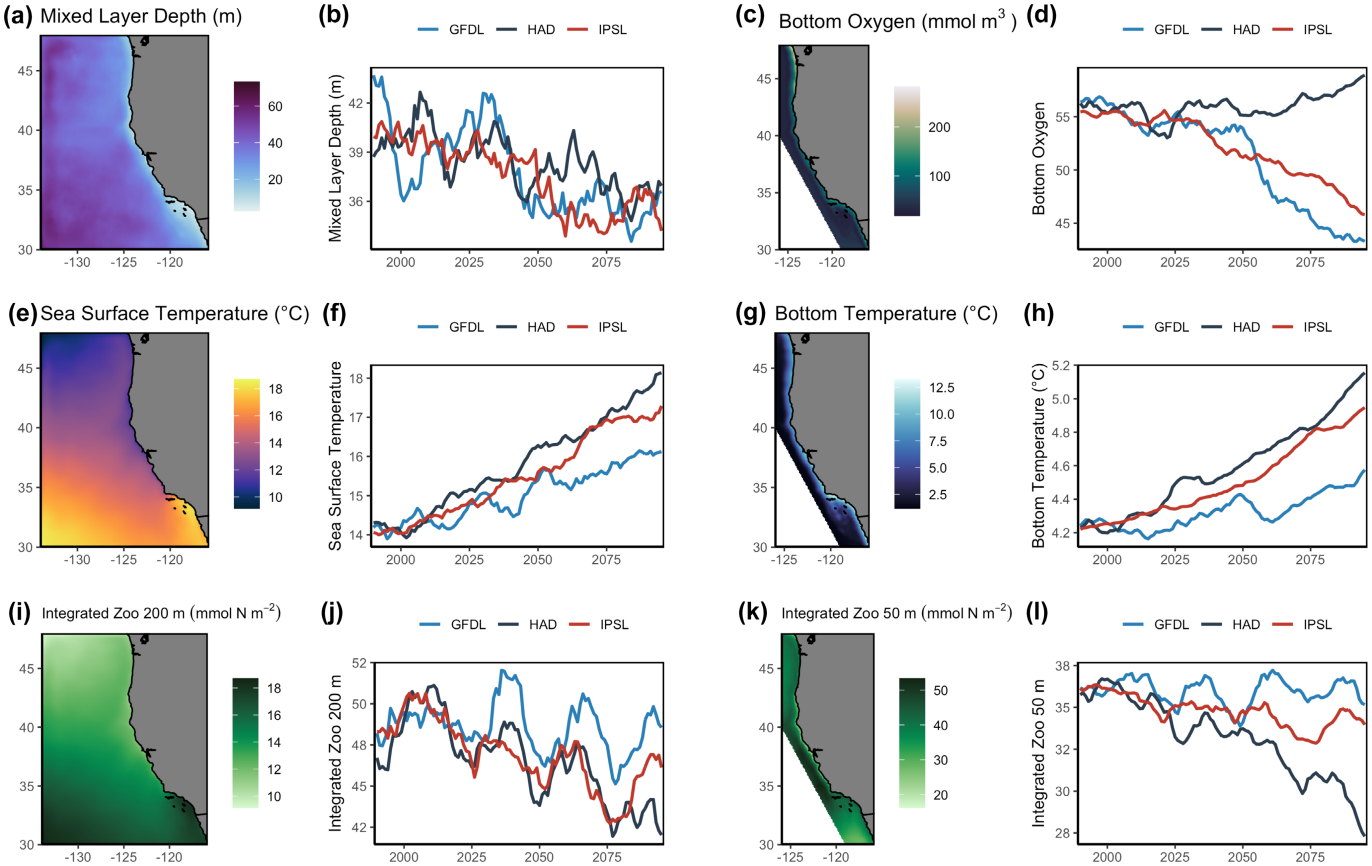
Maps and time series of dynamically downscaled environmental covariates projected to 2100. Maps (~10 km resolution) show the average historical spring conditions from 1985 to 2010 averaged across the downscaled HAD, GFDL, and IPSL earth system models (RCP8.5). Time series show the spatially averaged annual spring conditions (1985–2100) for each earth system model. The domain for (a, b) mixed layer depth, (e, f) sea surface temperature, and (i, j) 200 m integrated zooplankton reflects the ROMS extent, whereas the domain for (c, d) bottom oxygen, (g, h) bottom temperature, and (k, l) 50 m integrated zooplankton is limited to the inshore area to match species operating model domain. An 11‐year running mean is applied to the time series.

Environmental covariates used in species distribution simulations were obtained from regional ocean projections (Pozo Buil et al., 2021) forced by three ESMs from phase 5 of the Coupled Model Intercomparison Project (CMIP5) archive: Geophysical Fluid Dynamics Laboratory (GFDL) ESM2M, Hadley Center HadGEM2‐ES (HAD), and Institut Pierre Simon Laplace (IPSL) CM5A‐MR. These ESMs, hereafter referred to as GFDL, HAD, and IPSL, span the approximate range of potential changes in physical and biogeochemical conditions across all CMIP5 models (Pozo Buil et al., 2021). ESMs were downscaled using the Regional Ocean Modelling System (ROMS) coupled with a biogeochemical model (NEMUCSC) (Fiechter et al., 2018, 2021) based on the North Pacific Ecosystem Model for Understanding Regional Oceanography (NEMURO) (Kishi et al., 2007). The ROMS domain spans the CCS from 30 to 48°N and from the coast to 134°W at 0.1° horizontal resolution with 42 terrain‐following vertical layers (Figure 2). Each downscaled ESM used the Representative Concentration Pathway (RCP) 8.5 climate change scenario. While we only examined RCP 8.5, it should be noted that using RCPs 2.6 and 4.5 would result in only minor differences in the spread of future environmental change for the variables and ESMs examined here. Specifically, uncertainty in biogeochemical change among the chosen ESMs in RCP8.5 envelops the uncertainty among RCPs 2.6 and 4.5; while for temperature GFDL and HAD represent opposite ends of the spectrum for the projected magnitude of warming in the CMIP5 ensemble (Drenkard et al., 2021; Pozo Buil et al., 2021). As such, we do not explore scenario uncertainty. Environmental covariates used in species distribution simulations were sea surface temperature (SST; C), bottom temperature (BT; C), bottom oxygen (BO; mmol m^−3^), mixed layer depth (MLD; m), surface chlorophyll *a* (Chl‐*a*; mg m^−3^), and zooplankton concentration integrated over 50 m (zoo_50; mmol N m^−2^) and 200 m (zoo_200; mmol N m^−2^). These environmental covariates were averaged over spring months (March–May) annually (1985–2100) to encompass the seasonal period when ocean productivity is most influential on the long‐term population dynamics of most marine fishes in the CCS.

### 2.3 Operating models: Simulated species biomass

Biomass distributions for three species archetypes were simulated on the ROMS grid for each year and each ESM from 1985 to 2100. Simulations were run using the “virtualspecies” R package (Leroy et al., 2016) that is specifically designed to reflect real‐world ecological properties and species–environment relationships (Meynard et al., 2019). We refer to these simulated species distributions as “operating models.” Species simulations used a two‐step process. First, habitat suitability was calculated based on environmental data and specified species' habitat preferences (Table S1). Environmental preferences used to force species distributions varied among species archetypes based on representative life histories (see Supplementary Material). The domain for the HMS archetype was set to the entire CCS, whereas the CPS and GFS archetypes were reduced to inshore waters to reflect the CPS archetype's preference for pelagic waters over the continental shelf and slope, and the GFS archetype's preference for demersal shelf and slope habitats (Leeuwis et al., 2019; Stierhoff et al., 2020).

Second, total habitat suitability was calculated and converted to presence–absence using a logistic function (which specifies at what suitability value the species becomes present). When species were present, biomass was estimated from a log‐normal distribution, and when species were absent, biomass was set to zero. Biomass at each grid cell was multiplied by habitat suitability of that same grid cell to provide habitat‐informed biomass. For CPS and GFS archetypes, an additional biomass multiplier was used to encompass population‐level dynamics (Figure S1; see Supplementary Methods) (Punt et al., 2016). Specifically, CPS biomass was made to reflect boom‐bust population dynamics that are common in CPS species in the CCS, while GFS biomass integrated a 20‐year phase shift between low and high recruitment, as has been observed for sablefish (Haltuch et al., 2019). Simulated data were generated for each grid cell (HMS = 21,912 grid cells; CPS & GFC = 4012 grid cells) once per year for 116 years (1985–2100). Detailed methods for the simulation are provided in the Supplementary Material, and R code is provided on github (https://github.com/stephbrodie1/Projecting_SDMs).

### 2.4 Estimation models: Species distribution models

We parameterized a series of SDMs to estimate the relationship between simulated species biomass and covariates (Figure 1). Because these are fitted to data from an operating model, we refer to these SDMs as “estimation models.” Multiple approaches were tested to explore how decisions about model type and parameterization influence model accuracy and predictive performance (Brodie et al., 2020). We used four types of SDMs: generalized additive models (GAM), generalized linear mixed models (GLMM), boosted regression trees (BRT), and multilayer perceptron models (MLP; a type of artificial neural network model) (Table S2). Parameterization options included various combinations of environmental (E), spatial (S), and temporal (T) covariates (Figure 1; see Supplementary Methods). Spatial and temporal covariates can act as proxies for unobserved or unmeasured processes that drive species distributions and were included here given their common use in SDMs (typically called spatiotemporal models) (Brodie et al., 2020). We expect spatiotemporal SDMs with no environmental covariates to perform poorly over the projection period. We constructed all SDMs as delta (hurdle) models, where the probability of occurrence (binomial) and positive biomass (log‐normal) was estimated as separate processes. All SDMs were trained on data from 1985 to 2010, where only 500 random samples per year (2% of available data) were used for fitting (*n* = 13,000). Random samples included both presence and absence sampled across the entire domain. No SDM validation or model selection was required as our simulation experiment is designed to explore a range of model parameterizations.

Fitted SDMs were then used to predict species biomass on projected environmental data, for every year and grid cell in the domain. Only 500 randomly sampled grid cells per year (2011–2100) were used for testing purposes (*n* = 45,000), to match the resolution of samples used to train models. Importantly, not all environmental covariates used to simulate species biomass (see 2.3 above) were included in the fitted SDMs. Specifically, we used chlorophyll *a* as a proxy for prey fields (zooplankton) to approximate real‐world conditions where imperfect information is available for estimating species' habitat preferences. In addition to the 15 SDM parameterizations listed in Figure 1, we examined SDMs that only contained a single covariate of temperature (either surface or bottom temperature depending on the archetype). This experiment was done to test how underparameterized models that miss key environmental drivers of species distributions performs, and the degree to which this approach decreases model fit and increases projection uncertainty. We refer to these SDMs as “temperature‐only” models (Figure 1).

### 2.5 Model evaluation and uncertainty partitioning

We analyzed the SDMs using three metrics. First, we evaluated SDM performance using Spearman correlation coefficients between observed and estimated species biomass at each grid cell for each year. Second, we compared SDM performance to the level of environmental novelty experienced across each species' study domain. That is, over the projected period, we assessed the percent to which the multivariate environmental niche extrapolates relative to the niche defined in the historical fitting period (1985–2010). The multivariate environmental niche was calculated from every grid cell using the “compute_extrapolation” function in the *dsmextra* R package (Bouchet et al., 2020) based on the covariates used in SDMs, namely SST and MLD for the HMS archetype; SST, Chl‐*a*, and bathymetry for the CPS archetype; and BT, BO, and bathymetry for the GFS archetype. Novel habitat includes both single variable extrapolation and multivariate extrapolation (referred to as combinatorial extrapolation). We note that this method can provide a conservative estimate of novelty (Smith et al., 2022). This consideration of environmental novelty allows our results to be based on the relative degree of environmental extrapolation from historical conditions.

Finally, we partitioned the influence of uncertainty in species biomass predictions among ESMs, SDM type, and SDM parameterization. We do this using a dominance analysis, where we fit a linear model with annual species biomass predictions as the response variable, with ESMs (*n* = 3 factor levels), model type (*n* = 4 factor levels), and parameterization (*n* = 6 factor levels) as predictor covariates. We then apply the fitted linear model to the “dominanceAnalysis” function in the *dominanceanalysis* R package (version 2.0.0) (Navarrete & Soares, 2020) to determine the relative importance of each predictor covariate. We apply this approach for species biomass predictions across three regions in the CCS: north (>40°), central (34.5–40°), and south (<34.5°). We also apply this approach for the species biomass predictions across the whole domain and for the temperature‐only SDMs.

## 3 RESULTS

### 3.1 Environmental variability and simulated species distributions

**FIGURE 3:**
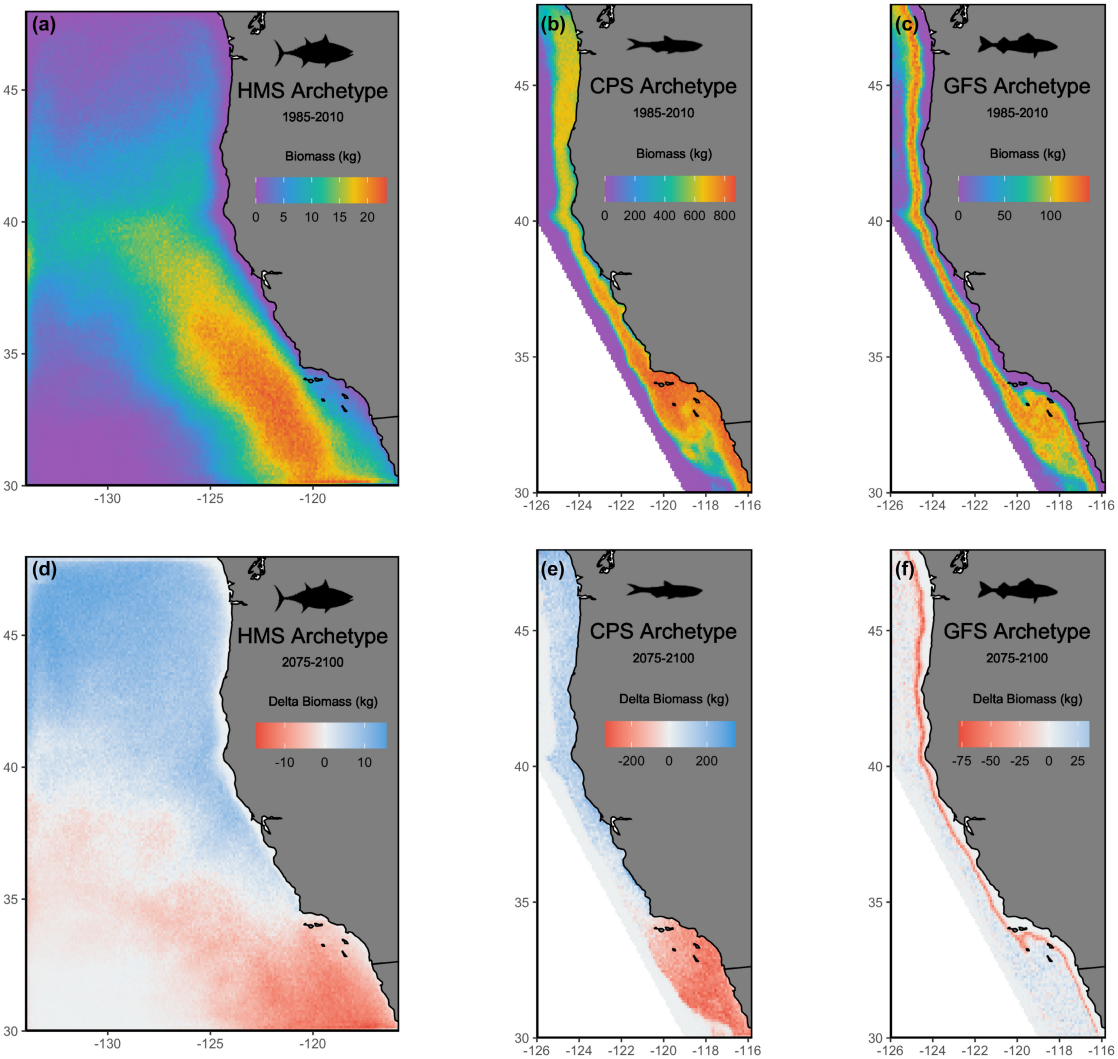
Simulated biomass distributions for highly migratory (HMS), coastal pelagic (CPS), and groundfish (GFS) species archetypes averaged from 1985 to 2010 (a–c) and the spatially explicit difference (future minus historical) in biomass averaged from projections for 2075–2100 (d–f). All results are averaged across earth system models.

Environmental variables showed strong spatial structure under future change scenarios, with coastal areas in particular having greater differences among ESMs (Figure 2). In general, surface and bottom temperature increased over the projection time period, while mixed layer depth, bottom oxygen, and zooplankton concentration decreased, with the latter two variables diverging substantially across earth system models (Figure 2d,j,l). Mixed layer depth and zooplankton concentration also showed strong decadal variability (Figure 2b,j,l). Simulated species biomass, which integrates these environmental covariates based on species habitat preferences (i.e., the operating models), also showed strong spatial patterns. The HMS archetype was more abundant in southern and offshore waters, CPS was largely restricted to inshore coastal waters, and GFS was more abundant in bottom shelf habitats (Figure 3a–c). When biomass distributions of each species were projected under the three ESMs, the HMS and CPS lost biomass in the southern area and gained biomass in the northern area of the domain, while the GFS lost biomass along the shelf break across the entire coastline (Figure 3d–f). Time series of biomass trends across the whole domain showed no clear trend in directionality (Figure S2), with trends reflecting the divergence among ESMs (Figure 2). For example, decadal trends in HMS biomass (Figure S2) relate to decadal variability in zooplankton concentration; CPS biomass under HAD declines (Figure S2) which aligns with the decrease in HAD zooplankton concentration (Figure 2l); while cyclical trends in GFS relate to the recruitment dynamics integrated in the species operating model (Figure S2).

### 3.2 How does the use of environmental and spatiotemporal covariates influence SDM projection accuracy?

**FIGURE 4:**
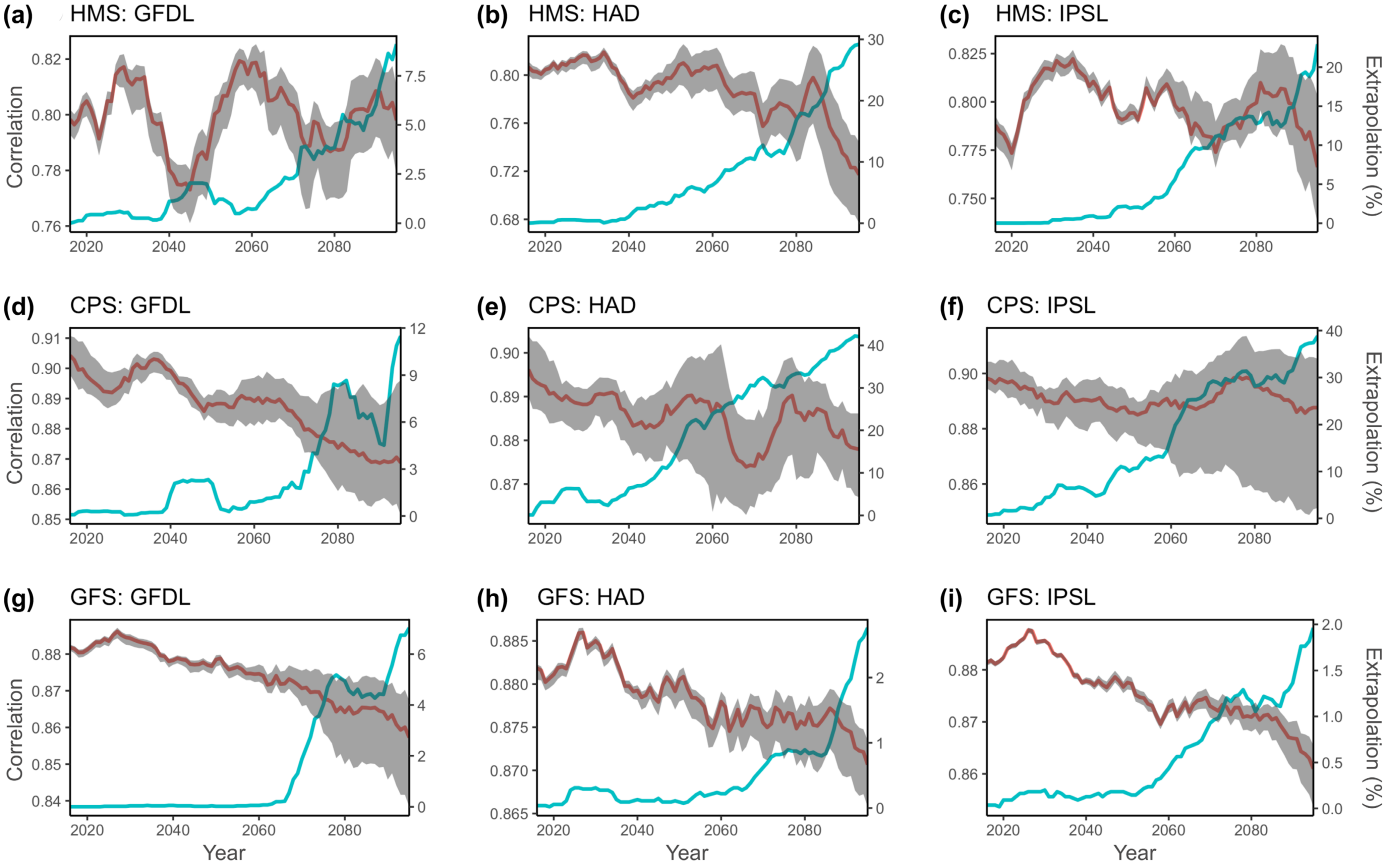
Annual correlation coefficient between simulated and estimated biomass (red line is ensemble mean) for three species archetypes (HMS: highly migratory species (a–c); CPS: coastal pelagic species (d–f); GFS: groundfish species (g‐i)) and three earth system models (HAD, GFDL, IPSL). Blue line shows the percent of environmental extrapolation experienced by SDMs, with extrapolation relative to the 1985–2010 training period. The ensemble mean of 12 estimation models is shown in red (ensemble mean does not include three SDMs that were considered to have poor performance over the projection period: GAM_S, GAM_EST, GLMM_ST). Grey shading indicates the maximum and minimum correlations from the 12 estimation models, and an 11‐year running mean was applied to the correlation and extrapolation time series. Note y‐axes differ among plots.

**FIGURE 5:**
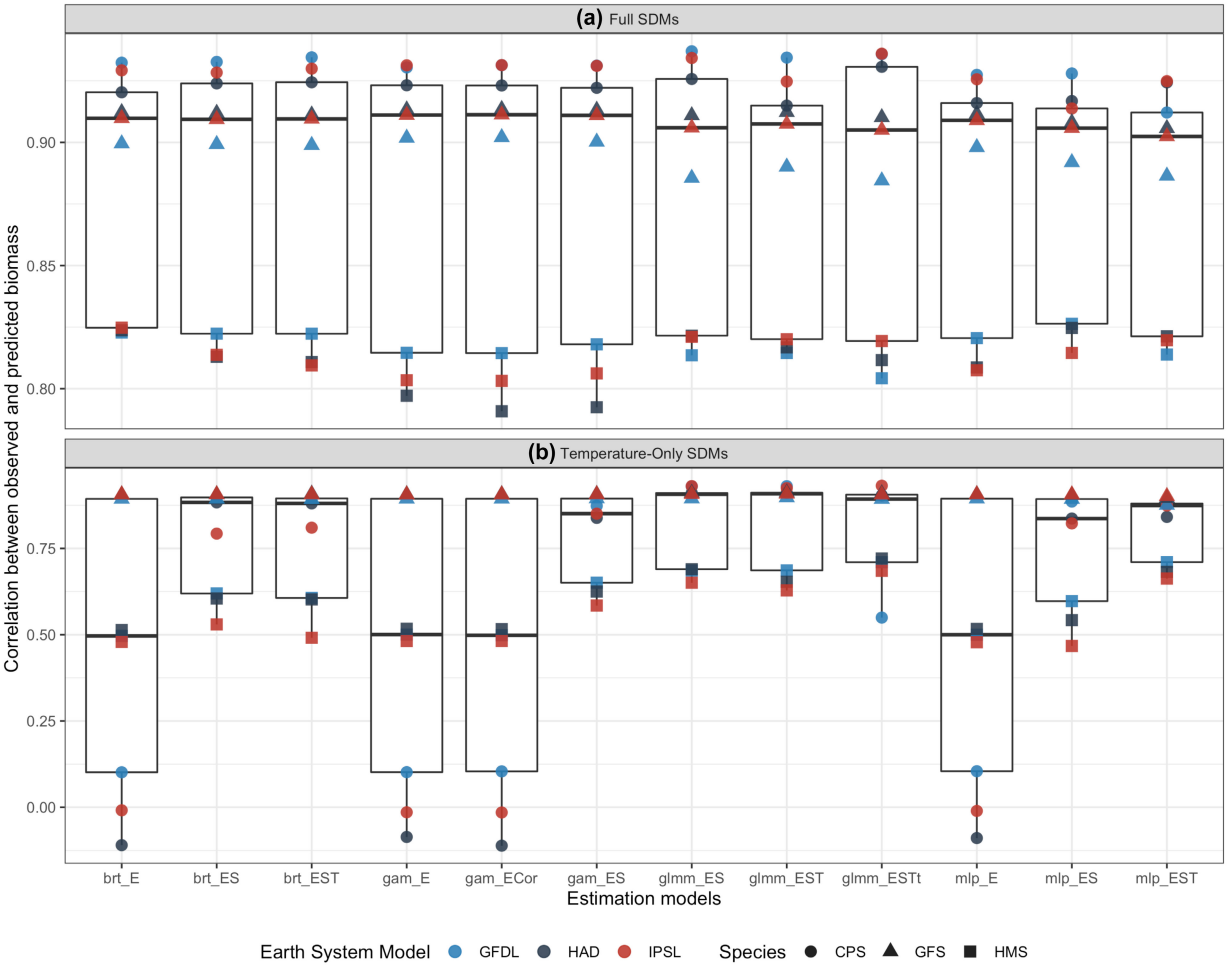
Correlation coefficients between simulated and estimated biomass for each species distribution model (a), showing loss of performance in the temperature‐only experiment (b). Correlations were calculated for projection period only (2011–2100). Colors represent the three earth system models, while symbols representing the three species archetypes. The ensemble mean across SDMs is shown. See Table S2 for description of SDMs. Note different y‐axis in each plot.

We tested 15 SDMs to evaluate the relative performance of model type and parameterization in projecting future biomass. Most SDMs accurately fit training data (1985–2010; correlations >0.77; Figure S3) and showed no spatiotemporal biases in fit (Figure S5), the sole exception was the spatial‐only GAM (GAM_S). Similar patterns were seen for the probability of the presence component of the delta SDMs, as measured by area under the receiver operating curve (AUC) values (Figure S4). Model projection performance varied with the covariates included and the SDM structure, generally decreasing over the projection period and with greater spread among SDMs (Figure 4). Including spatial covariates in addition to environmental covariates when projecting SDMs helped to improve performance over the projection period, particularly when the species had a strong and persistent spatial structure to their distribution (CPS and GFS archetypes) or when the SDM did not capture the dominant mechanisms driving distributions (e.g., temperature‐only SDM) (Figure 5). SDMs that included spatial covariates but did not include environmental covariates (GAM_S and GLMM_ST) had poor performance over the projection period and were removed from subsequent analyses (Figures S3 and S4). Including temporal covariates when projecting SDMs did not inhibit performance over the projection period, except for the spatiotemporal GAM in which the temporal component was extrapolated and led to poor predictive performance (Figures S3 and S4) and high within‐model error (Figure S6; GAM_EST was removed from subsequent analyses). Semiparametric (GAM) and mixed‐effects (GLMM) SDMs required appropriate specification of temporal correlation in spatial effects to perform well, whereas machine learning techniques (BRTs and MLPs) performed well without explicit incorporation of temporal and spatial interactions (Figures S3 and S4).

### 3.3 How does SDM performance degrade over the projection period?

Prediction performance of SDMs degraded progressively over the forecast period (Figure 4). Decreased prediction performance was more pronounced in the GFS and CPS archetypes, likely reflecting the increased uncertainty introduced from the underlying population dynamics (i.e., boom‐bust dynamics for CPS, and recruitment feedback for GFS) integrated into the operating model that were not captured in the SDMs. Despite the long‐term trend of decreasing model performance, there was substantial decadal variability in SDM performance for the HMS and CPS archetypes (Figure 4a–e), which reflects similar decadal patterns seen in mixed layer depth and zooplankton concentration (Figure 2a,j,l).

### 3.4 How do SDMs perform when predicting to novel environmental conditions?

Multivariate environmental conditions became increasingly novel over the projection period, where by 2100, 21% of the modeled domain (mean across ESMs and species) had environmental conditions not previously experienced in data used to fit each species SDM (1985–2010) (Figure 4; Figure S7). This degree of environmental extrapolation experienced by SDMs varied among species archetypes, with limited extrapolation seen for GFS (4% of the data used to project SDMs was extrapolated by 2100) compared with HMS (28%) and CPS (32%) by 2100 (Figure 4; Figure S7). Across species archetypes and ESMs, we found that SDM performance generally decreased as extrapolation increases— that is, SDMs perform worse in more novel climates (Figure 4). We also found that the spread of projected biomass estimates became increasingly wider as environmental novelty increased (Figure 4). Interestingly, there was limited decadal variability evident in environmental novelty (Figure S7), yet decadal variability was evident in the environmental covariates (Figure 2) and in the model performance (Figure 4).

### 3.5 What are the dominant sources of uncertainty and how do they change over the projection period?

**FIGURE 6:**
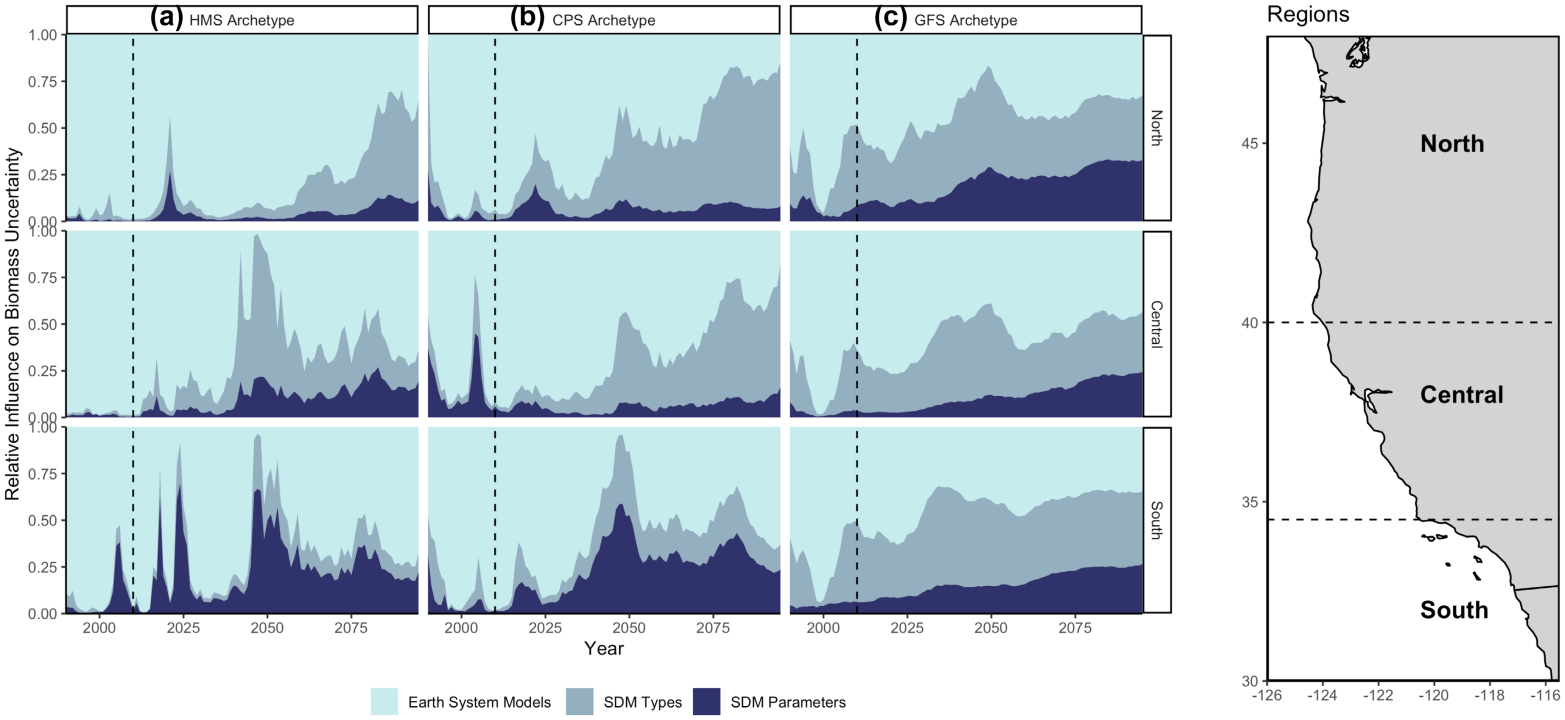
Relative uncertainty in biomass predictions for each region (north, central, south) and species archetype: (a) HMS, (b) CPS, (c) GFS. Uncertainty is partitioned across earth systems models, SDM type, and SDM parameterization. Dashed vertical line indicates when projections start. An 11‐year running mean was applied. Map on the right shows regions of the California current system.

**FIGURE 7:**
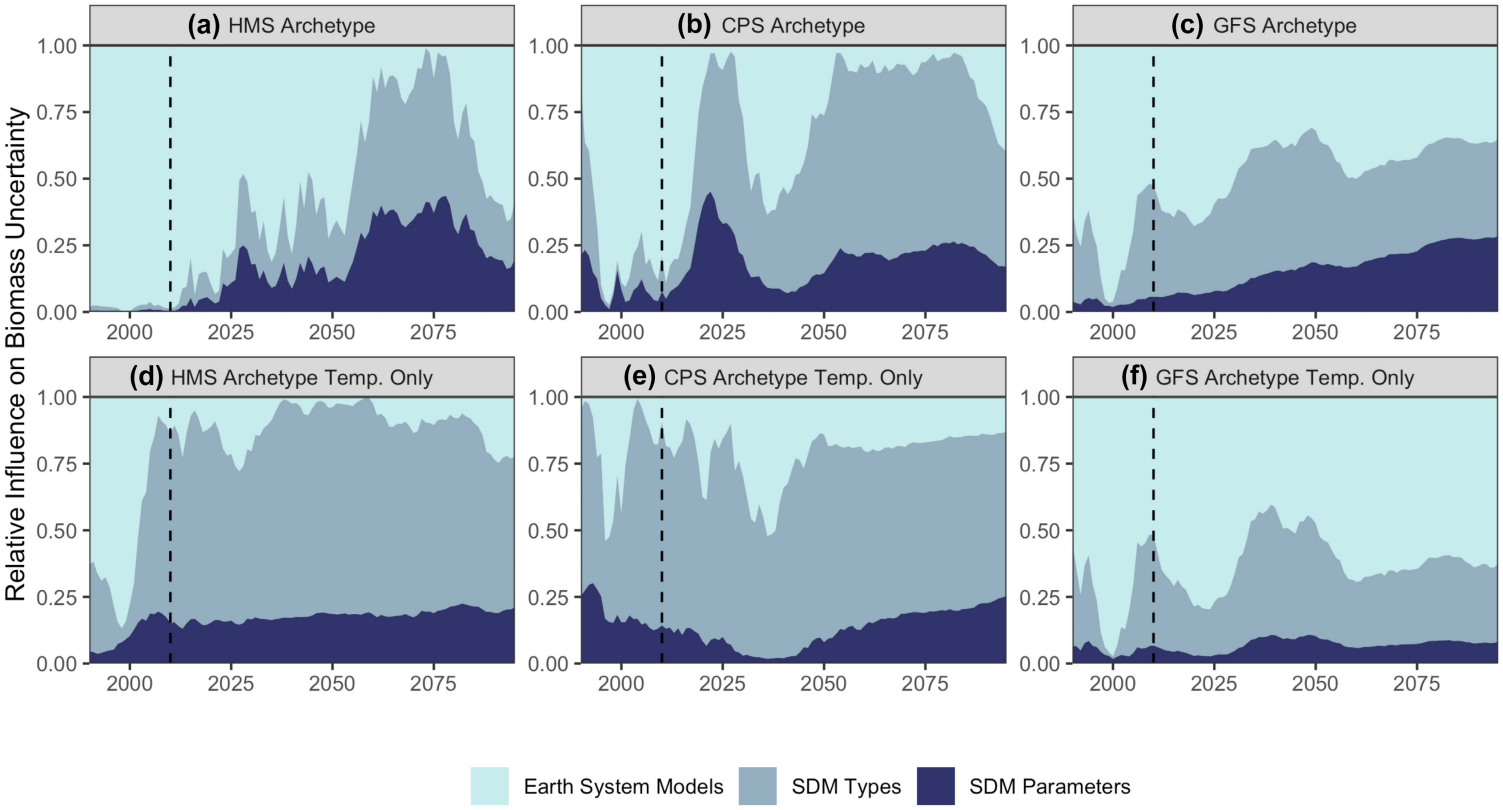
Relative uncertainty in biomass predictions for each species archetype (integrating the three regions in Figure 6), for SDMs parameterized with all environmental variables (a–c), and with temperature only (d–f). Uncertainty is partitioned across earth systems models, SDM type, and SDM parameterization. Dashed vertical line indicates when projections start. An 11‐year running mean was applied.

We found that uncertainty in SDM biomass projections increased over the 90‐year projection period and could exceed uncertainty among ESMs (Figures 6 and 7). The contribution of SDM uncertainty to total uncertainty is highest in the northern region of the CCS across all species (Figure 6). This reflects an environmental signal, where projected conditions increasingly diverge in the central and southern regions compared to the northern region (Figure S8). SDM uncertainty has the capacity to dwarf ESM uncertainty when SDMs have incomplete information or are mis‐specified (Figure 7), highlighting the important contribution SDM structure makes to uncertainty in long‐term projections. The relative importance of each type of uncertainty is influenced by the ecological processes underlying our species archetypes. For instance, HMS distributions were simulated to respond primarily to the environment, and therefore uncertainty was driven by oceanographic variables from the ESM which results in HMS having higher ESM uncertainty compared to other archetypes (Figure 6a). GFS and CPS were simulated to have distributions constrained by bathymetry and population dynamics, where SDM type and parameterization tended to drive uncertainty, particularly over the long term (Figure 6b,c). In general, the dynamics in uncertainty partitioning appear to relate primarily to divergence among ESM projections and the magnitude of extrapolation to novel environmental conditions experienced by SDMs.

## 4 DISCUSSION

Climate change has already caused the geographic redistribution of many marine species, resulting in conflicts across jurisdictional boundaries and creating challenges for resource managers (Holsman et al., 2019; Liu & Molina, 2021; Palacios‐Abrantes et al., 2022; Pinsky et al., 2018). Realistic projections of potential future ecological states can help prepare resource managers for different scenarios of climate change and ecological redistribution (Hollowed et al., 2020). However, there is a need to quantify uncertainty across SDMs and ESMs, and how uncertainty within these models propagates over time. By quantifying the performance and uncertainty of SDMs when projected over climate forecast horizons, we identify relationships between environmental novelty and ecological model performance, as well as quantify the contribution of SDM uncertainty to climate projection uncertainty. Below we discuss our findings and provide recommendations for projecting SDMs, and summarize our discussion in Box 1.

BOX 1 **Recommendations for quantifying and reducing uncertainty in climate projections of species distributions. See the discussion section for full details and justification**

**Quantifying Uncertainty**

1. Studies projecting species distribution models (SDM) should pay increased attention to quantifying and communicating uncertainty to assist end users in scenario planning.
2. Using a combination of multiple diverging earth system models and multiple types or parameterizations of SDMs will effectively capture uncertainty.
3. Quantify uncertainty over time, as SDM uncertainty can dominate at longer time horizons (>40 years), and decadal variability can drive nondirectional trends in uncertainty.

**Reducing Uncertainty**

1. Spend effort on reducing SDM extrapolation rather than improving model fit (e.g., fitting species data over a broader range of environmental conditions).
2. Consider including spatial covariates (e.g., latitude), especially for species with strong spatial structure to their distributions or when SDMs do not capture dominant mechanisms driving distributions.
3. Carefully explore the addition of temporal covariates (e.g., year) to SDMs, and suggest they be integrated within artificial intelligence models, or within models that can incorporate spatiotemporal variation.

### 4.1 Characterizing uncertainty

Quantifying uncertainty is critical to ensuring appropriate communication of climate scenarios and anticipated impacts for marine ecosystems to better prepare stakeholders and communities for change (Tittensor et al., 2021). Furthermore, comparing the magnitude of multiple sources of uncertainty can help determine where to invest effort to increase the precision of projections most efficiently. We found that uncertainty among a series of well‐fit and similarly performing SDMs can exceed uncertainty generated across ESMs, and that this result was consistent across the archetypal species explored. Notably, while the differences in performance among SDMs were relatively minor, SDM model type (i.e., BRTs, GAMs, MLPs, and GLMMs) was the major source of uncertainty in projections, particularly at longer time horizons (>40 years). The increases in SDM uncertainty over time are partly due to the emergence of no‐analog climates which force model extrapolation. Our results validate the findings of other studies that have explored the relative contribution of SDM uncertainty to projections (e.g., Morley et al., 2020; Reum et al., 2020; Thuiller et al., 2019)—but importantly, the consistency in results occurs despite our SDMs having a much better model fit and predictive performance than typical empirical SDMs. This suggests that simply improving the fit and performance of correlative models may not help to reduce SDM projection uncertainty, especially when extrapolation is likely. Future work could test the performance of additional model types and response variables, such as species presence models like Maxent (Phillips & Dudík, 2008). Indeed, next‐generation modeling techniques, such as shape‐constrained models (Citores et al., 2020), hybrid SDMs that explicitly integrate mechanistic or process‐explicit responses (Briscoe et al., 2019; Evans et al., 2016), or approaches that account for non‐stationarity in ecological responses may be required (Bueno de Mesquita et al., 2021; Malick et al., 2020) but first need to be tested as to whether that can improve extrapolative performance. These next‐generation techniques can require subjective choices or large amounts of data, posing challenges and trade‐offs for modelers interested in projecting species distribution dynamics (e.g., Briscoe et al., 2019; Fordham et al., 2018).

Our simulation study is able to explicitly identify some of the main mechanisms that lead to increased projection uncertainty—particularly model extrapolation into novel environmental space and differences in habitat preferences across species archetypes. While our analysis provides valuable insight into the contribution of SDM uncertainty, it is an overly simplistic assessment (by design) that likely underestimates the true total uncertainty that would be seen with empirical data. Interestingly, our approach attributed more uncertainty to SDMs than what has been shown in empirical studies (Morley et al., 2020; Thuiller et al., 2019) despite our models being better fit than typical empirical SDMs. However, we anticipate that decreased model fit and predictive performance associated with empirical data would act to increase the uncertainty within and among SDMs, and decrease the time horizon when SDM uncertainty exceeds ESM uncertainty (and we show this with the temperature‐only simulation). We also show that SDM uncertainty was more dominant for the GFS and CPS archetypes whose distribution was additionally constrained by nondynamic variables and whose overall biomass trend had some imposed temporal structure independent of environmental drivers. As we begin to build multispecies projections of ecosystem response to future climate change (Fulton et al., 2011; Tittensor et al., 2021), we may need to explore several axes of uncertainty based on the characteristics of the projected species. We note that projected environmental change in the study region was not sufficient to drive any of our species archetypes to a biomass approaching zero (Figure S2). In studies where future conditions are likely to exceed environmental tolerance limits of a species, and lead to local extirpation, model uncertainty can decrease through time as environmental conditions become increasingly unfavorable.

### 4.2 Recommendations for SDM projection studies and practitioners

Our simulation framework was designed to test the accuracy of projected SDMs. Our results inform the following recommendations for SDM projection studies, including recommendations for how to reduce and communicate uncertainty in future studies (see also Box 1). Our first recommendation is to carefully consider the inclusion of spatial covariates in SDMs and the underlying mechanistic processes they represent. Including spatial covariates improved predictive performance for all three species archetypes, and for all four model types (GAM, GLMM, BRT, and MLP). In particular, adding spatial covariates was particularly useful when species had strong spatial structure to their distributions and environmental covariates were not able to capture the dominant mechanisms driving distribution (e.g., temperature‐only models for CPS and GFS archetypes). However, for many mobile species, there may be a point at which historical spatial relationships begin to break down and no longer accurately predict species distributions, thus care should be taken when interpreting projected SDMs that contain these spatial structures and perhaps supplement model evaluations with expert opinion and guidance (e.g., Warren et al., 2020). Indeed, Barnes et al. (2022), showed more complex SDMs improved model fit but failed to skillfully forecast species distributions. Furthermore, our results indicated that not capturing the appropriate mechanisms driving species distributions can lead to poor model performance when projecting. However, we did see decadal variability in SDM performance which related to cyclical trends in projected zooplankton concentration, as compared to the directional trend in temperature typically seen in climate projections. This leads to a need to think more creatively about which other covariates or processes could be derived or measured that are currently beyond what our standard instrumentation and ocean models allow, such as subsurface environmental data or prey fields (e.g., Brodie, Jacox, et al., 2018; Goodman et al., 2022; Tolimieri et al., 2018).

Our second recommendation is to consider ways to reduce the extent to which models extrapolate, so as to help reduce model uncertainty over the projection period. Our results indicate that simply improving the fit of correlative models may not reduce SDM projection uncertainty, but rather effort would be better spent trying to sample data and understand species responses over a broader range of environmental conditions. This could be achieved by collecting more empirical data, particularly at the range edges of species distributions or beyond historical boundaries of sampling surveys, conducting lab‐based experiments to better understand species thresholds, or explicitly integrating mechanistic or process‐explicit species responses. For highly mobile species in particular, building SDMs with data from a larger geographic range than the region of interest for projections may help to avoid truncating species response curves and improve SDM performance over the projection period (Brodie, Litherland, et al., 2018; Guisan & Thuiller, 2005).

Each model we tested differs in how it is fit to historical data and how it extrapolates on novel data. BRTs extrapolate by predicting a constant biomass at the value of the “nearest” terminal node (i.e., biomass remains largely static in novel environments). MLPs can extrapolate beyond the training data, but this is often not recommended by practitioners due to potentially unrealistic results (Gardner & Dorling, 1998). GAMs use a spline to fit data, and will extrapolate from data under a specified derivative penalty (typically penalizing a non‐linear shape) (e.g., Riutort‐Mayol et al., 2020). Conversely, GLMMs extrapolate based on a linear combination of fixed‐effect terms and the estimated temporal correlation of spatial fields (when such effects are included). The GLMMs in this study were more parametrically structured than the other model types, yet did not provide any additional inference or predictive performance. We primarily attribute this to the lesser flexibility of covariate responses, compared to the nonlinear splines, trees, or neural networks seen in other model types, and potential effects of spatial fields and spatially autocorrelated covariates. We acknowledge that the simulation framework may have unfairly considered GLMMs given that more flexible responses (e.g., splines) can be incorporated into such models and additional model structure can better help to resolve complex ecological processes (Barnes et al., 2022; Barnett et al., 2021). Our results indicate that the impact of extrapolation on model performance is difficult to predict, and more research is needed on methods for measuring and improving extrapolation, and the trade‐offs between resolving ecological processes and more accurately defining response curves (e.g., Brodie et al., 2020).

We note that adding annual temporal covariates did not provide any additional improvements in SDM predictive capacity, but rather was capable of significantly degrading performance (e.g., GAM_EST). In the case of GAM_EST, the annual trend in the spatial components is extrapolated for 90 years, which unsurprisingly becomes inaccurate. There are many ways to include and constrain (see Supplementary Material) temporal covariates, and our simulation framework only examined annual time steps. For species with strong subannual phenological patterns, or that respond to an unknown spatiotemporal process, parameterization of a temporal covariate may be beneficial (Brodie et al., 2020; Tolimieri et al., 2018). Also, if temporal covariates are important for improving model fits to observations, then there is a strong case for including or constraining them in projections. We recommend careful exploration of temporal covariates and suggest they be integrated within an artificial intelligence model, or within models that can incorporate spatiotemporal variation with appropriate assumptions regarding the nature of temporal correlation of spatial patterns.

Our final recommendation is to ensure that future studies projecting SDMs pay increased attention to quantifying and communicating uncertainty. SDM projection studies present a range of plausible futures to assist ocean stakeholders and resource managers with scenario planning and adaptation strategies. Inherent to this exercise is capturing and communicating realistic uncertainty to assist end users in scenario planning. Future work could focus on examining uncertainty derived from observation uncertainty (e.g., imperfect sampling of the system), or process error variability arising from fitting SDMs. Our results highlight that using a combination of both ESMs and SDMs is an effective approach to capturing realistic uncertainty, and we recommend that future studies consider a similar approach.

Our recommendations are based on a simplified simulation framework, designed to test the accuracy of projected SDMs. Our SDMs fit the simulation data well, and in general were better fit than studies using empirical data. Specifically, mean *R* ^2^ for the HMS archetype was .63 while Muhling et al. (2019) published an albacore CPUE SDM with an *R* ^2^ of .31. The same pattern was seen for occurrence in the anchovy CPS archetype (AUC of 0.99 vs. 0.83) (Muhling et al., 2019) and sablefish groundfish archetype (AUC 0.79 vs. 0.71–0.73 in the Gulf of Alaska) (Pirtle et al., 2019). There are many ecological processes that we have not captured in our simulation that are known to influence species distributions (e.g., density dependence, interspecific interactions, life history, population structure, endothermy, recruitment dynamics, etc.), and further work could focus on integrating such structuring processes into simulations (Grimmett et al., 2021) or empirical studies (Jaatinen et al., 2021). Additionally, ecological impacts due to climate change will not just limited to species movements. Climate‐induced changes to trophic structure, animal physiology, and species interactions will all increase the uncertainty and unpredictability of projections, and has not been captured in this analysis. Comparison of residuals between simulated SDMs and empirical SDMs would be helpful to make inference on how to better build operating models with real‐life data generating processes. Overall, our results likely underestimate absolute uncertainty, but still provide informative results based on a best‐case scenario, and highlight trends in partitioned uncertainty. Because our simulations were based on empirical species with real environmental variability and our results presented on a relative scale, our recommendations are generalizable to empirical data of mobile marine species and to other regions outside of the CCS.

This study arose from a workshop in support of the Western Regional Action Plan of the NOAA Fisheries Climate Science Strategy (Busch et al., 2016; Link et al., 2015). The workshop highlighted additional next steps and areas of high priority research to prepare for and mitigate climate impacts on eastern North Pacific fisheries, managed and protected species, and habitats. These next priority steps include testing the utility of various SDM performance metrics (e.g., range edges, climate velocity, habitat displacement) as a means to accurately quantify and communicate climate impacts on species distributions; test the performance of hybrid SDMs that incorporate mechanistic and process‐explicit responses (Briscoe et al., 2019); compare the near‐term projections of simulations to those based on empirical data for specific species (e.g., albacore, anchovy, and sablefish) as a means to explicitly prepare regional resource managers for climate impacts to key fisheries species; explore the role of interspecific interactions (predation, competition) on projections (Tekwa et al., 2022); and examine the changes in fishing and other human responses to changes in species availability (Selden et al., 2020; Smith et al., 2021). On the US West Coast, these efforts will inform scenario planning activities conducted by the Pacific Fisheries Management Council (PFMC) under its Climate and Communities Initiative (https://www.pcouncil.org/documents/2020/11/scenarios‐for‐west‐coast‐fisheries‐climate‐and‐communities‐initiative.pdf/), and the PFMC scenarios can be aligned to assumptions about biophysical dynamics to inform additional evaluation of SDM performance. Future research that addresses these priority next steps will help to increase the production, delivery, and use of climate‐related information required by resource managers and other ocean stakeholders.

## 5 CONCLUSION

Understanding species distributions is a key aspect of developing climate‐resilient fisheries, and SDMs can capture some of the drivers of ecological change to help inform robust management strategies (Karp et al., 2019). Ecological projections of species distributions can be used in scenario planning exercises for resource managers, and our results highlight the important role SDM modeling decisions have in contributing to projection uncertainty. Specifically, we find that uncertainty from ESMs will dominate over the next several decades, but that will eventually be exceeded by uncertainty from SDMs. Results were consistent across species archetypes, but notably were moderated depending on the underlying dynamics driving species distributions (i.e., environmental variability and population dynamics) and the extent to which novel environmental conditions forced SDMs to extrapolate. Climate‐resilient fisheries management benefits from qualitative perspectives, for instance in risk assessment, and from quantitative dynamic and adaptive approaches that forecast and manage for ecosystem shifts over a range of timescales (Holsman et al., 2019). Our results are able to accurately capture SDM performance over climate projections and communicate the contribution of SDM uncertainty to ecological projections. This is a critical result that can help resource managers understand the “known unknowns,” and that increased uncertainty is likely under empirical scenarios.

## CONFLICT OF INTEREST

The authors declare no conflicts of interest.

## Supporting information


Data S1

## Data Availability

Code to reproduce simulated species projections is available on github: https://github.com/stephbrodie1/Projecting_SDMs. Monthly downscaled climate projections, and simulated species distributions and model predictions are available on dryad (https://doi.org/10.7291/D1JQ2K).
