# Supplementary material for: Recommendations for quantifying and reducing uncertainty in climate projections of species distributions
Source: Glob Chang Biol. 2022 Aug 17;28(22):6586–601. doi: 10.1111/gcb.16371 (PMC9805044; doi:10.1111/gcb.16371)
Supplement: Supplementary file 1 — Data S1 [file GCB-28-6586-s001.pdf]

Supplementary Material

Recommendations for quantifying and reducing uncertainty in climate projections of species distributions

Stephanie Brodie*[1,2], James A. Smith[1,3], Barbara A. Muhling[1,3], Isaac C. Kaplan[4], Lewis A.K. Barnett[5], Gemma Carroll[6], Paul Fiedler[3], Steven J. Bograd[1,2], Elliott L. Hazen[1,2], Michael G. Jacox[1,2,7], Kelly S. Andrews[4], Cheryl L. Barnes[8], Lisa Crozier[4], Jerome Fiechter[1], Alexa Fredston[9], Melissa A. Haltuch[4], Chris J. Harvey[4], Elizabeth Holmes[4], Melissa A. Karp[10], Owen R. Liu[4], Michael J. Malick[4], Mercedes Pozo Buil[1,2], Kate Richerson[4], Christopher N Rooper[11], Jameal Samhouri[4], Rachel Seary[1,2], Rebecca L. Selden[12], Andrew R Thompson[3], Desiree Tommasi[1,3], Eric J. Ward[4], Rich Zabel[4]

[1] Institute of Marine Sciences, University of California Santa Cruz, Monterey, CA, USA
[2] Environmental Research Division, Southwest Fisheries Science Center, National Marine Fisheries Service, National Oceanic and Atmospheric Administration, Monterey, CA, USA
[3] Southwest Fisheries Science Center, National Marine Fisheries Service, National Oceanic and Atmospheric Administration, San Diego, CA, USA
[4] Northwest Fisheries Science Center, National Marine Fisheries Service, National Oceanic and Atmospheric Administration, Seattle, WA, USA
[5] Alaska Fisheries Science Center, National Marine Fisheries Service, National Oceanic and Atmospheric Administration, Seattle, WA, USA
[6] Environmental Defense Fund, Seattle, USA
[7] Physical Sciences Laboratory, Earth System Research Laboratories, National Oceanic and Atmospheric Administration, Boulder, CO, USA
[8] Cooperative Institute for Climate, Ocean, and Ecosystem Studies, University of Washington, Seattle, WA, USA
[9] Department of Ecology, Evolution, and Natural Resources, Rutgers University, New Brunswick, NJ, USA

[10] ECS Tech, *in support of,* NOAA Fisheries Office of Science & Technology, Silver Spring, MD
[11] Pacific Biological Station, Fisheries and Oceans Canada, Nanaimo, British Columbia, Canada
[12] Department of Biological Sciences, Wellesley College, Wellesley, MA, USA

*lead and corresponding author: sbrodie@ucsc.edu

Supplementary Material

Operating Models: Simulated Species Biomass

Spatial biomass for three species archetypes were simulated for each year and each ESM from 1985-2100. Simulations used the 'virtualspecies' R package (Leroy et al., 2016) that is spe-cifically designed to reflect real-world properties and datasets (Meynard et al., 2019). Species simulations used a two-step process. First, habitat suitability was simulated based on environmental data and defined species' habitat preferences (Table S1). The distributions used to specify these responses were normal (e.g. SST had a domed influence on habitat suitability), or logistic (e.g. prey probability of presence had a monotonically increasing influence on habitat suitability) (Table S1). Environmental variables used to force species distributions varied among species archetypes. For the HMS archetype, habitat suitability was forced by SST, MLD, and a simulated prey species, where the prey species was forced by SST and zoo_200. For the CPS archetype, habitat suitability was forced by SST, zoo_50, and bathymetry. For the GFS archetype, habitat suitability was forced by bottom temperature, bottom oxygen, and bathymetry. Bathymetry was used in both CPS and GFS simulations to help additionally structure known spatial distributions, where CPS prefer inshore waters and GFS prefer slope habitats (Leeuwis et al., 2019; Stierhoff et al., 2020). The domain for the CPS and GSF archetypes was reduced to inshore waters (inshore of 126°W) to reflect these preferences.

Second, habitat suitability was calculated and converted to species presence-absence using a logistic function. The parameters of this function are listed in Table S1, and define at what habitat suitability each species becomes present. This step used the 'generateSpFromFun' function in the 'virtualspecies' package. When species were present, biomass was estimated from a log-normal distribution, and when species were absent biomass was set to zero. Biomass at each grid cell was then multiplied by habitat suitability of that same grid cell to provide habitat-informed biomass. For CPS and GFS archetypes, an additional biomass multiplier was used to encompass population-level dynamics from 1985-2100 (Figure S1). For CPS, biomass was additionally multiplied by annual indices from a population model (Punt et al., 2016) to encompass boom-bust population dynamics that are common in CPS species in the CCS (e.g. sardine and anchovy). Further, in years when CPS biomass was below the 25% quantile ('bust' years; Fig S1), we additionally multiplied biomass by a latitudinal gradient to force a preference towards southern areas. This preference for southern waters during years of low population biomass is seen in Anchovy population dynamics (MacCall et al., 2016). For GFS, biomass was additionally multiplied by an annual index that reflected a 20-year phase shift between low and high recruitment, as seen for sablefish (an example groundfish species) (Haltuch et al., 2019). Additionally, the biomass habitat suitability multiplier was lagged by 2 years to reflect how prior habitat suitability can influence recruitment. Simulated data were generated for each grid cell (HMS = 21912 grid cells; CPS & GFC = 4012 grid cells) once per year for 116 years (1985-2100). R

code for the simulation is provided on github (https://github.com/stephbrodie1/Projecting_SDMs).

Estimation Models: Species Distribution Models

We fit a series of different SDM types and parameterization options to the simulated species biomass (Figure 1; Table S2). We used four types of SDMs: generalized linear mixed models (GLMM), generalized additive models (GAM), boosted regression trees (BRT), and multilayer perceptron (MLP) - a type of neural network model. Parameterization options included various combinations of environmental (E), spatial (S), temporal covariates (T) (Figure 1). All SDMs were delta models, where both the probability of presence (binomial with logit link) and biomass are modeled as individual components. Because biomass is skewed, we used log(biomass) as a response for the positive component of the model. All SDMs were trained on data from 1985-2010, where only 500 random samples per year were used for fitting (n=13 000). Random samples assumed a perfect probability of detecting the species. Fitted SDMs were then used to predict species biomass on projected environmental data. Only 500 random samples per year were used for testing purposes (n=45 000).

GAMs were fitted using the *mgcv* R package (Wood, 2017). Five separate GAMs were fitted for each species archetype, with each model parameterized differently (Table S2): environmental covariates only; spatial covariates only; both environment and spatial covariates; environmental, spatial, and temporal covariates; and environmental covariates with additional residual spatiotemporal correlation implemented with a Gaussian correlation structure from the *nlme* R package (Pinheiro et al., 2017). Environmental covariates and spatial covariates were included using a thin plate regression spline, with smoothness selected via Generalized Cross Validation (GCV) (Table S2). Spatiotemporal terms were either included as a 3-way tensor product smooth (predictions vary non-linearly in space and by year, with each year allowing for a differing spatial smooth), or as a Gaussian correlation structure (Table S2). Each of the GAM models represents a different approach to including spatial or temporal covariates, and there are different ways that these models propagate uncertainty into the future: uncertainty in the models with no time effects (GAM_E, GAM_S, GAM_ES) is not affected by time; the tensor smooth in model GAM_EST will extrapolate a time signal and tends to increase the prediction's standard errors over time; and the correlated errors in model GAM_Ecor model do not propagate through time. Extrapolating a time pattern 90 years (as done in GAM_EST) would generally be a risky decision, and an alternative is to make the time value constant by specifying a first-order penalty (m=1) for that variable, although this may over-constrain the model.

GLMMs were fitted using the sdmTMB R package (version 0.0.19.90) (Anderson et al., in review; Anderson, 2019). These methods differ from the GAM approach above in that they implement

the Stochastic Partial Differential Equation (SPDE) method to estimate Gaussian random fields as Gaussian Markov random fields (Lindgren et al., 2011). As with the GAMs described above, spatial or spatiotemporal fields may be added to any GLMM model. Models estimated with the SPDE approach differ in that they also provide estimates of spatial covariance and derived quantities (spatial range). Four separate GLMMs were fitted for each species archetype, with parameterization options (Table S2) including: environmental covariates and constant spatial effects implemented via a spatial random field; constant and time-varying spatial effects implemented via a spatial random field and spatiotemporal random fields with temporal correlation following a first order autoregressive (AR1) process (Anderson, 2019); environmental covariates and spatially varying temporal trends implemented via spatial random fields for the intercept and slope (Barnett et al., 2021); and environmental covariates, constant and time-varying spatial effects implemented via a spatial random field and AR(1) spatiotemporal random fields. All environmental variables were included as quadratic terms to allow for non-linear responses. Like the GAMs above, each of these alternative model configurations propagates future uncertainty differently. The model with only environmental and spatial effects (GLMM_ES) behaves similarly to the GAM_E model in that uncertainty does not increase through time. The GLMMs with a spatiotemporal component (GLMM_S, GLMM_EST) have uncertainty increasing over time (as with GAM_EST) because the spatiotemporal fields are modeled as AR(1). Model GLMM_EST deals with time slightly differently, in that the trend is estimated as a spatially varying coefficient; with this model, uncertainty is similar to the GLMM_ES or equivalent GAMs in that it is constant through time.

BRTs were fitted using the *dismo* package (Elith et al., 2008), with a tree complexity of 3, bag fraction of 6, and a learning rate of 0.01 or 0.001 to ensure >1000 trees during the fitting process. Three BRTs were fit for each species archetype, with parameterization options (Table S2) including: environmental covariates only; environmental and spatial covariates; and environmental, spatial, and temporal covariates.

MLPs were fitted using the *neuralnet* package (Fritsch et al., 2019) using the resilient backpropagation with weight backtracking algorithm and a logistic activation function. Three MLPs were fit for each species archetype, with parameterisation options (Table S2) including: environmental covariates only; environmental and spatial covariates; and environmental, spatial, and temporal covariates. MLPs used 3 neurons in the single hidden layer, and the threshold was adjusted between 0.2 and 0.5 to ensure convergence.

Supplemental References:

Anderson, S. C. (2019). *A reproducible data synopsis for over 100 species of British Columbia groundfish*. Canadian Science Advisory Secretariat (CSAS).

Anderson, S., Ward, E., English, P., & Barnett, L. (in review). *SdmTMB: an R package for fast, flexible, and user-friendly generalized linear mixed effects models with spatial and spatiotemporal random fields.* https://doi.org/10.1101/2022.03.24.485545

Barnett, L. A. K., Ward, E. J., & Anderson, S. C. (2021). Improving estimates of species distribution change by incorporating local trends. *Ecography*, *44*(3), 427–439. https://doi.org/10.1111/ecog.05176

Elith, J., Leathwick, J. R., & Hastie, T. (2008). A working guide to boosted regression trees. *Journal of Animal Ecology*, *77*(4), 802–813.

Fritsch, S., Guenther, F., & Guenther, M. F. (2019). Package 'neuralnet.' *Training of Neural Networks*.

Haltuch, M. A., Johnson, K. F., Tolimieri, N., Kapur, M. S., & Castillo-Jordán, C. (2019). Status of the sablefish stock in US waters in 2019. *Pacific Fisheries Management Council, Portland, OR*.

Leeuwis, R. H. J., Nash, G. W., Sandrelli, R. M., Zanuzzo, F. S., & Gamperl, A. K. (2019). The environmental tolerances and metabolic physiology of sablefish (Anoplopoma fimbria). *Comparative Biochemistry and Physiology Part A: Molecular & Integrative Physiology*, *231*, 140–148. https://doi.org/10.1016/j.cbpa.2019.02.004

Leroy, B., Meynard, C. N., Bellard, C., & Courchamp, F. (2016). Virtualspecies, an R package to generate virtual species distributions. *Ecography*, *39*(6), 599–607.

Lindgren, F., Rue, H., & Lindström, J. (2011). An explicit link between Gaussian fields and Gaussian Markov random fields: The stochastic partial differential equation approach. *Journal of the Royal Statistical Society: Series B (Statistical Methodology)*, *73*(4), 423–498.

MacCall, A. D., Sydeman, W. J., Davison, P. C., & Thayer, J. A. (2016). Recent collapse of northern anchovy biomass off California. *Fisheries Research*, *175*, 87–94.

Meynard, C. N., Leroy, B., & Kaplan, D. M. (2019). Testing methods in species distribution modelling using virtual species: What have we learnt and what are we missing? *Ecography*.

Pinheiro, J., Bates, D., DebRoy, S., Sarkar, D., Heisterkamp, S., Van Willigen, B., & Maintainer, R. (2017). Package 'nlme.' *Linear and Nonlinear Mixed Effects Models, Version*, *3*(1).

Punt, A. E., MacCall, A. D., Essington, T. E., Francis, T. B., Hurtado-Ferro, F., Johnson, K. F., Kaplan, I. C., Koehn, L. E., Levin, P. S., & Sydeman, W. J. (2016). Exploring the implications of the harvest control rule for Pacific sardine, accounting for predator dynamics: A MICE model. *Ecological Modelling*, *337*, 79–95.

Stierhoff, K. L., Zwolinski, J. P., & Demer, D. A. (2020). *Distribution, Biomass, and Demography of Coastal Pelagic Fishes in the California Current Ecosystem During Summer 2019 Based on Acoustic-Trawl Sampling*.

Wood, S. N. (2017). *Generalized additive models: An introduction with R*. CRC press.

Table S1 Variables used to simulate species spatially-explicit biomass and their parameter values associated with a distribution. All variables define habitat suitability responses, except for 'Biomass' which is the argument used to determine abundance when present, and 'Occurrence' which is the function used to convert habitat suitability to a presence-absence. Three species archetypes are shown: Highly Migratory Species (HMS), Coastal Pelagic Species (CPS), and Groundfish species (GFS).

**Species Archetype**	**Name**	**Description**	**Parameter 1**	**Parameter 2**	**Distribution**
HMS	SST (°C)	Sea surface temperature	μ = 17	σ = 4	normal
	MLD (m)	Mixed layer depth	μ = 50	σ = 30	normal
	Prey presence	Preference for prey	α = -0.15	β = 0.4	logistic
	Prey SST (°C)	Prey sea surface temperature	μ= 14	σ = 7	normal
	Prey Zoo200 (mmol N m^{-2})	Zooplankton integrated over top 200m	α= -10	β= 45	logistic
	Biomass (kg)	Biomass if species considered present in a grid cell	log μ = 3.29	log (σ) = 0.26	Log normal
CPS	SST (°C)	Sea surface temperature	μ = 16	σ = 6	Normal
	Zoo50 (mmol N m^{-2})	Zooplankton integrated over top 50m	α = -5	β = 20	Logistic
	Bathymetry (m)	Spatial covariate	α = -500	β = -2000	Logistic
	Biomass (kg)	Biomass if species considered present in a grid cell	log μ = 6.87	log (σ) = 0.14	Log normal
GFS	BT (°C)	Bottom temperature	μ = 4	σ = 3	Normal
	BO (mmol m^3)	Bottom oxygen	μ = 57	σ = 62	Normal
	Bathymetry (m)	Spatial covariate	μ = 900	σ = 1600	Normal
	Biomass (kg)	Biomass if species considered present in a grid cell	log μ = 5.14	log (σ) = 0.22	Log normal
All species	Occurrence (0 or 1)	Occurrence as a function of habitat suitability	α = -0.7	β= 0.4	Logistic

Table S2 Summary of model configurations and parameterization in R syntax for an example species archetype, highly migratory species (HMS). Environmental variables for the HMS archetype include sea surface temperature (SST), surface chlorophyll-a (Chla), and mixed layer depth (MLD). SDMs include boosted regression trees (BRT), multilayer perceptron (MLP), generalized additive model (GAM), and generalized linear mixed model (GLMM). SDM parameterization options include combinations of environmental (E), spatial (S), temporal covariates (T)

**Model**	**Description**	**R syntax**
BRT_E	Environmental covariates only	gbm.x = SST, Chla, MLD
BRT_ES	Environmental and spatial covariates	gbm.x = SST, Chla, MLD, lat, lon
BRT_EST	Environmental, spatial, and temporal covariates	gbm.x = SST, Chla, MLD, lat, lon, year
MLP_E	Environmental covariates only	SST + Chla + MLD
MLP_ES	Environmental and spatial covariates	SST + Chla + MLD + lat + lon
MLP_EST	Environmental, spatial, and temporal covariates	SST + Chla + MLD + lat + lon + year
GAM_E	Environmental covariates only	s(SST) + s(Chla) + s(MLD)
GAM_S	Spatial covariates only	s(lat,lon)
GAM_ES	Environmental and spatial covariates	s(SST) + s(Chla) + s(MLD) + s(lat,lon)
GAM_EST	Environmental, spatial, and temporal covariates	s(SST) + s(Chla) + s(MLD) + te(lat,lon,year)
GAM_Ecor	Environmental covariates with additional residual spatiotemporal correlation	s(SST) + s(Chla) + s(MLD); correlation=corGaus(form=~lat+lon|fYear)
GLMM_ES	Environmental covariates and a spatial random field	SST + Chla + MLD + SST^2 + $Chla^2$ + MLD^2 ; spatial = “on”, spatiotemporal = “off”
GLMM_ST	Spatial and AR1 spatiotemporal random fields	spatial = “off”, spatiotemporal = “AR1”
GLMM_EST	Environmental covariates, and spatial and AR1 spatiotemporal random fields	SST + Chla + MLD + SST^2 + $Chla^2$ + MLD^2 ; spatial = “off”, spatiotemporal = “AR1”
GLMM_ESTt	Environmental covariates, a spatial random field, and spatially varying temporal trend random field.	SST + Chla + MLD + SST^2 + $Chla^2$ + MLD^2; spatial = "on",<br>spatiotemporal = "off",<br>spatial_varying = ~ 0 + year

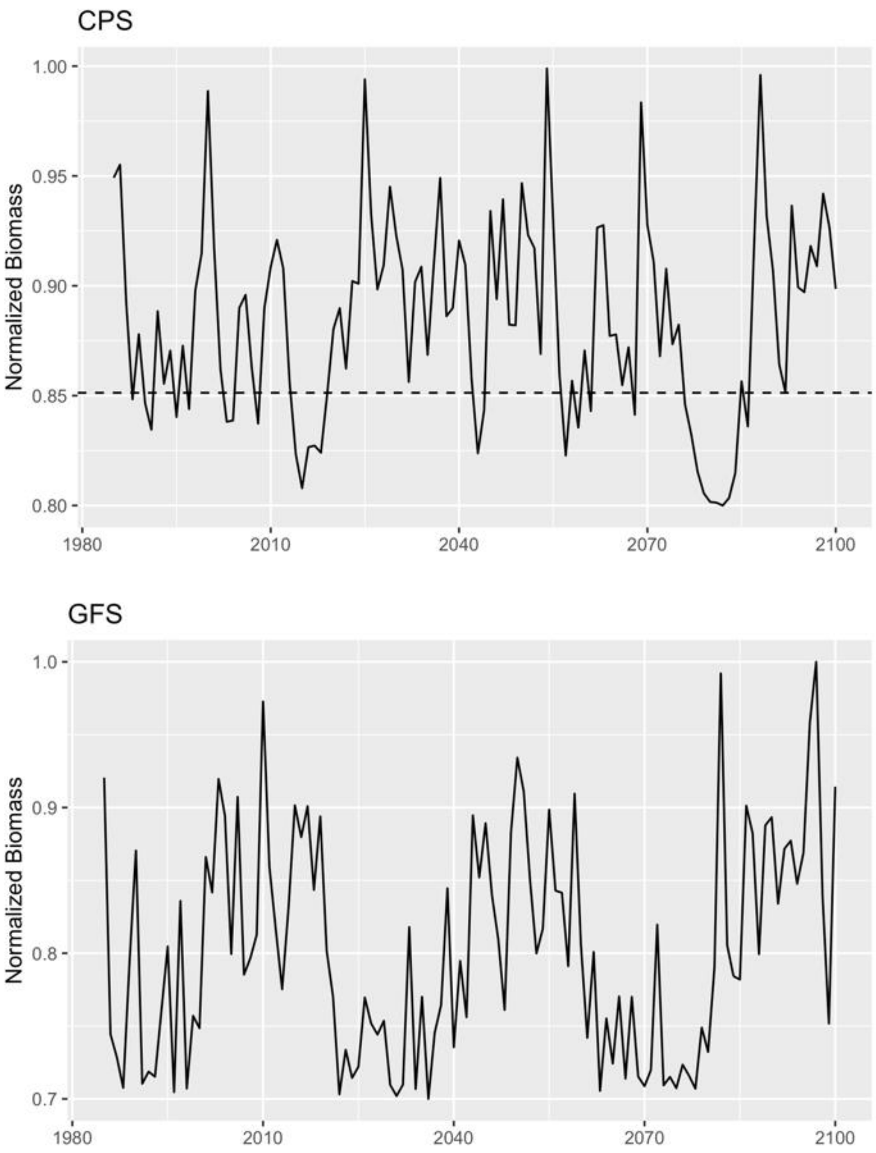

Figure S1 Time-series of normalized biomass for CPS and GFS archetypes. Annual indices are used as a biomass multiplier to encompass population trends in the species simulations. CPS reflects boom-bust dynamics from a population model (Punt et al., 2016), and the dashed line indicates the 25% quantile threshold used to force a preference of southern habitats when biomass is low. GFS reflects 20-year phase shifts of recruitment which impacts total population biomass.

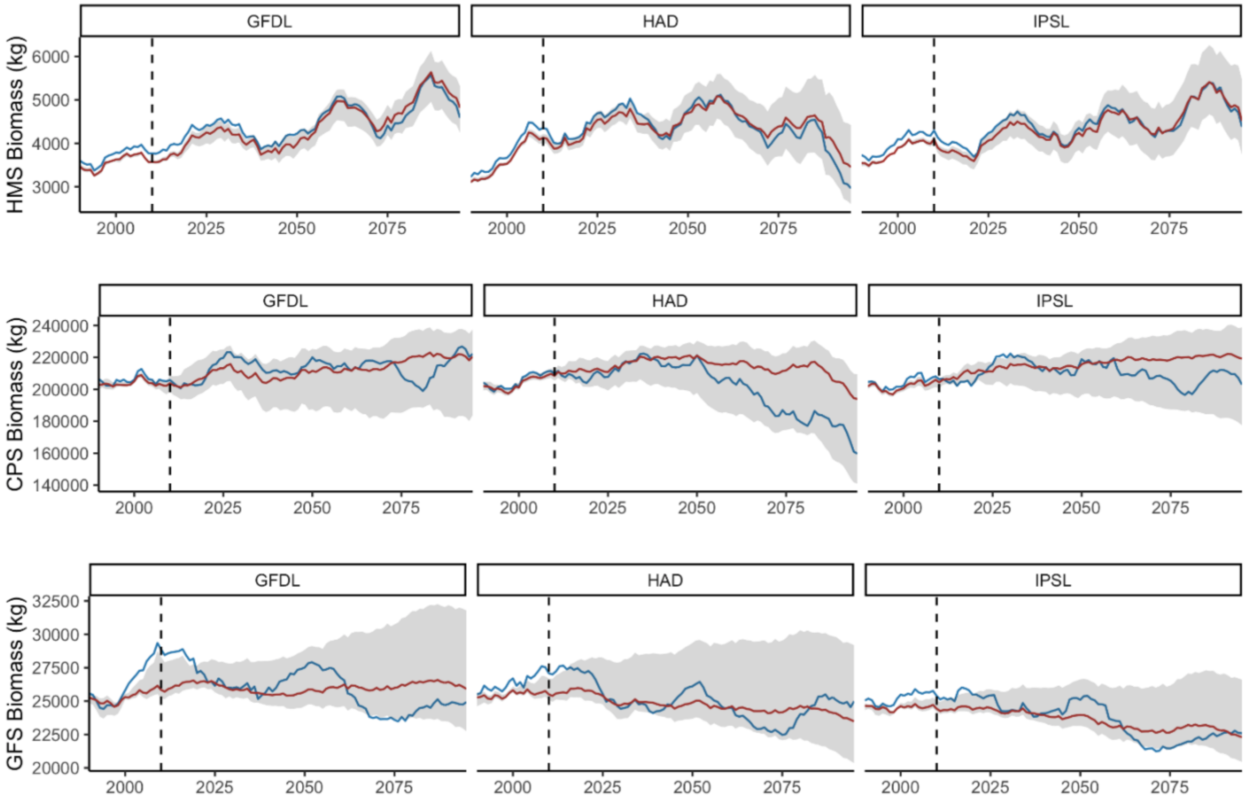

Figure S2 Time-series of simulated (blue) and estimated (red) species biomass for each earth system model. Red line indicates the ensemble mean, with gray shading showing the spread across 12 species distribution models. An 11-year running mean is applied to the time-series.

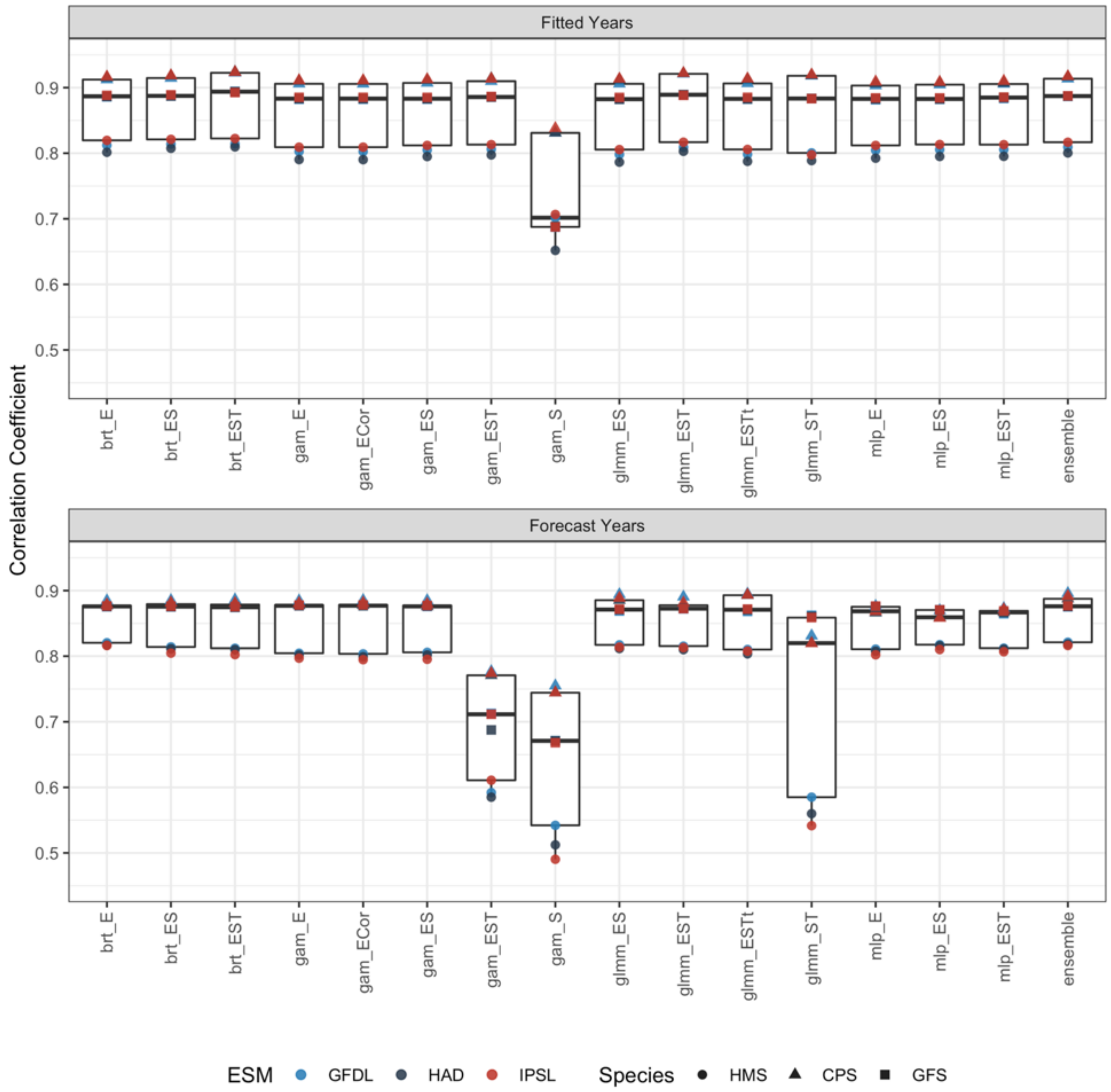

Figure S3 Spearman correlation coefficient between simulated and estimated biomass for each SDM on fitted data (top; 1985-2010) and forecast (bottom; 2011-2100) data. Results for each species archetype (symbol) and earth system model (color), and the ensemble mean across SDMs are shown.

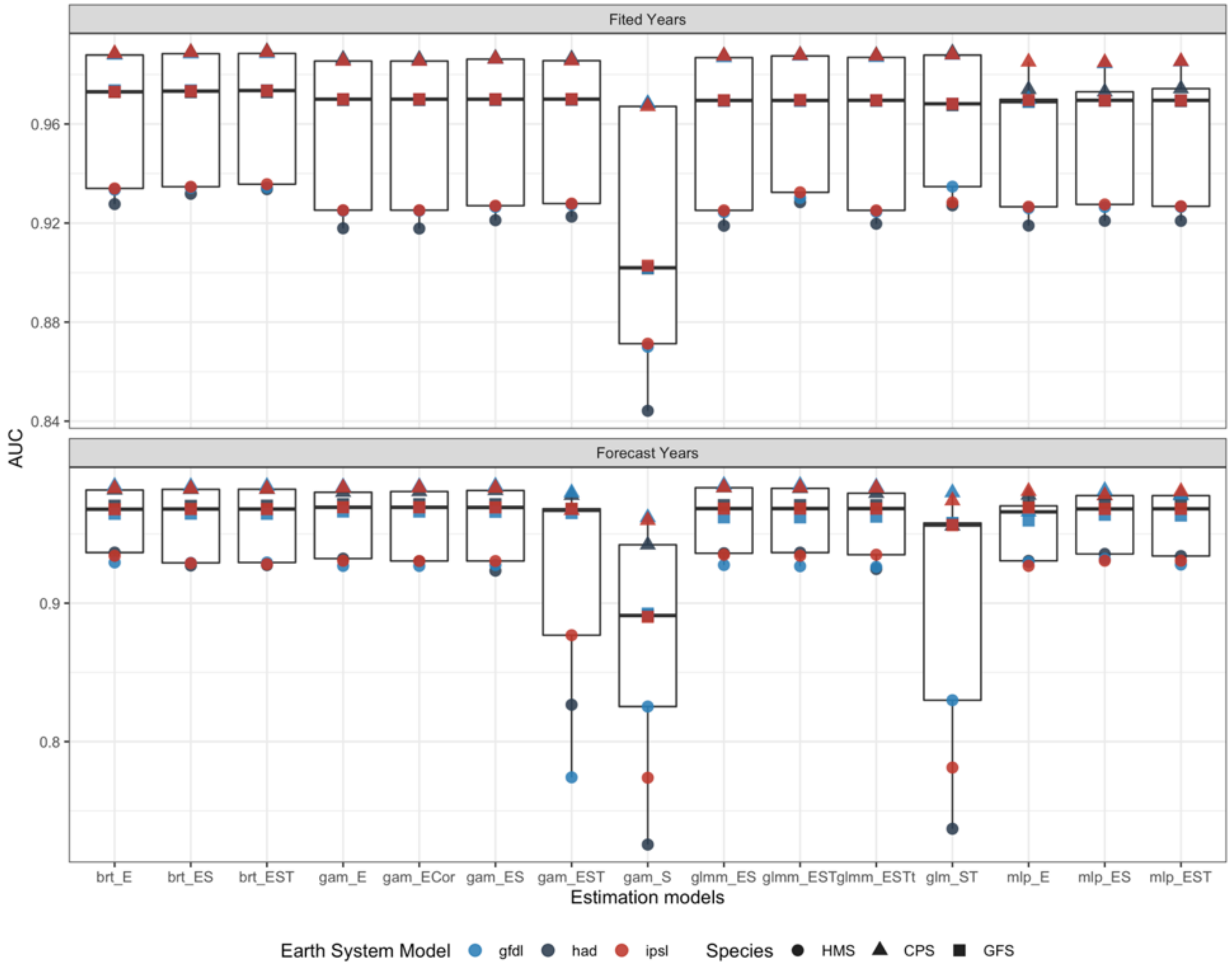

Figure S4 Area under the receiver operating curve (AUC) between simulated and estimated habitat suitability for each SDM on fitted data (top; 1985-2010) and forecast (bottom; 2011-2100) data. Results for each species archetype (symbol) and earth system model (color) are shown.

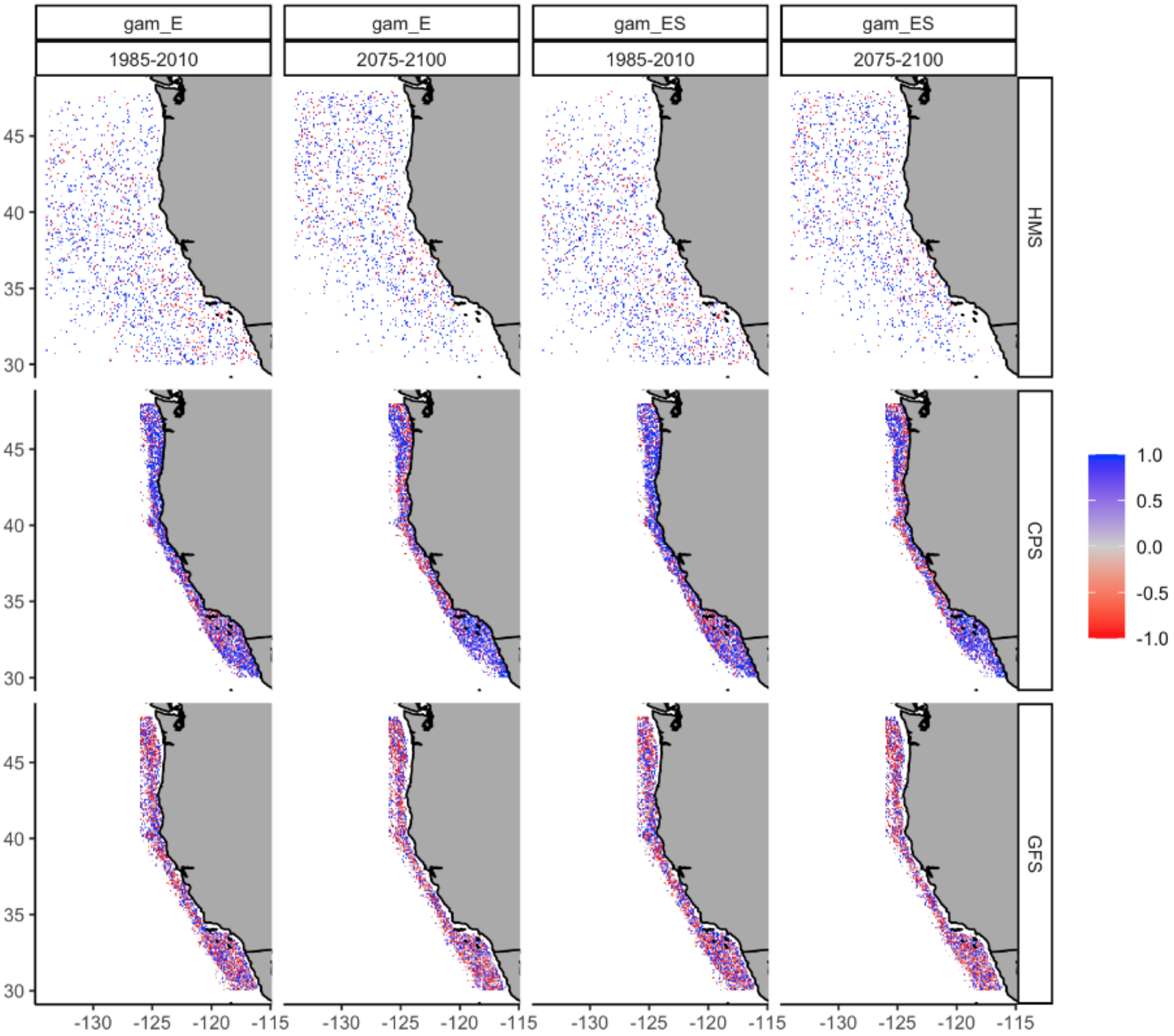

Figure S5 Spatial correlation coefficients between simulated and estimated (GAM_E and GAM_ES models) biomass for the three species archetypes, averaged across historical (1985-2010) and future (2075-2100) periods. Results from the HAD earth system model are shown.

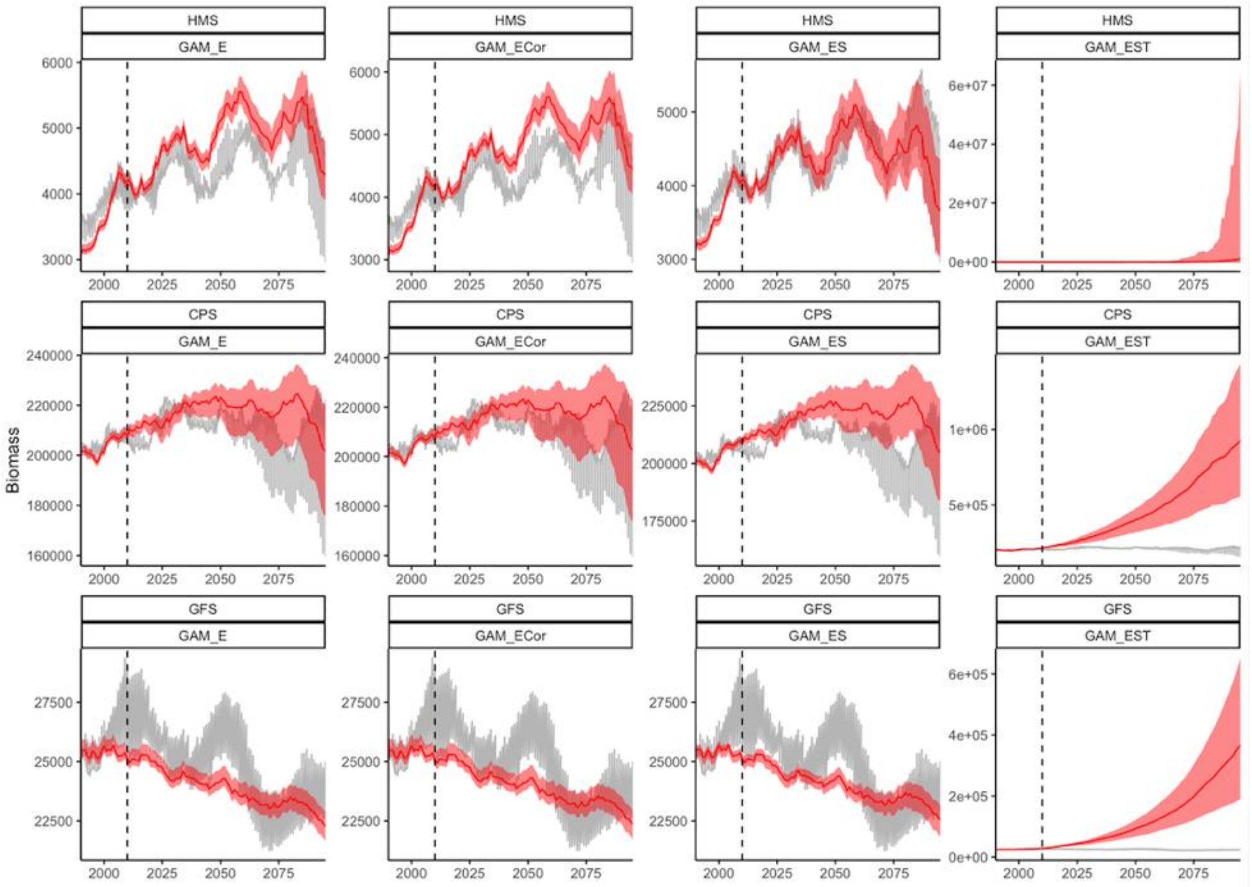

Figure S6 Time-series of simulated (grey line) and estimated (red line) biomass for species archetypes and the HAD earth system model. Within-model uncertainty for GAM SDMs (GAM_E; GAM_ECor; GAM_ES; GAM_EST) was generated from 100 samples from the posterior distribution of fitted models, with mean indicated by the red line and range of estimates indicated by the red shading. An 11-year running mean was applied.

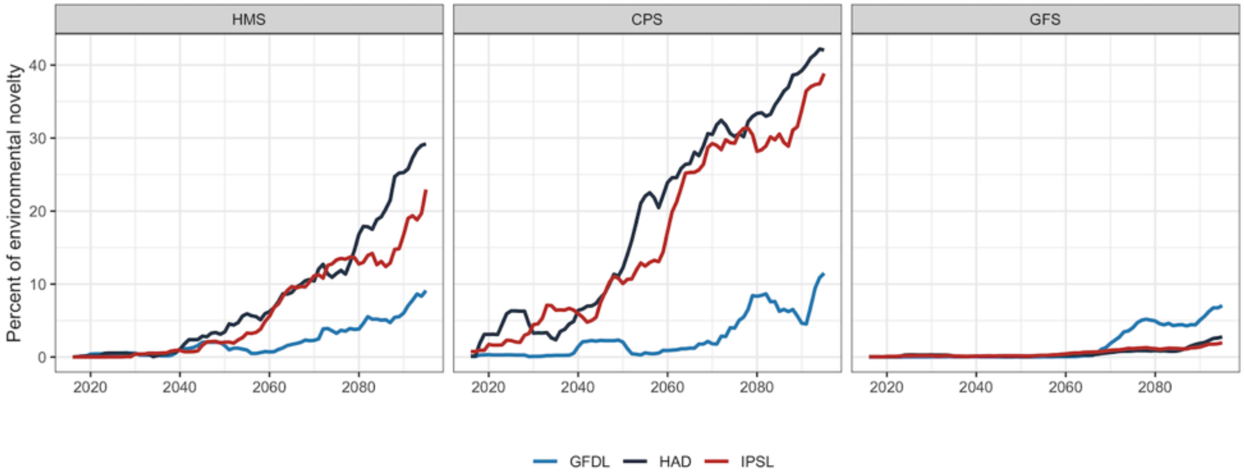

Figure S7 Percent of environmental extrapolation experienced by SDMs, with novelty relative to the 1985-2010 training period. An 11-year rolling mean was applied.

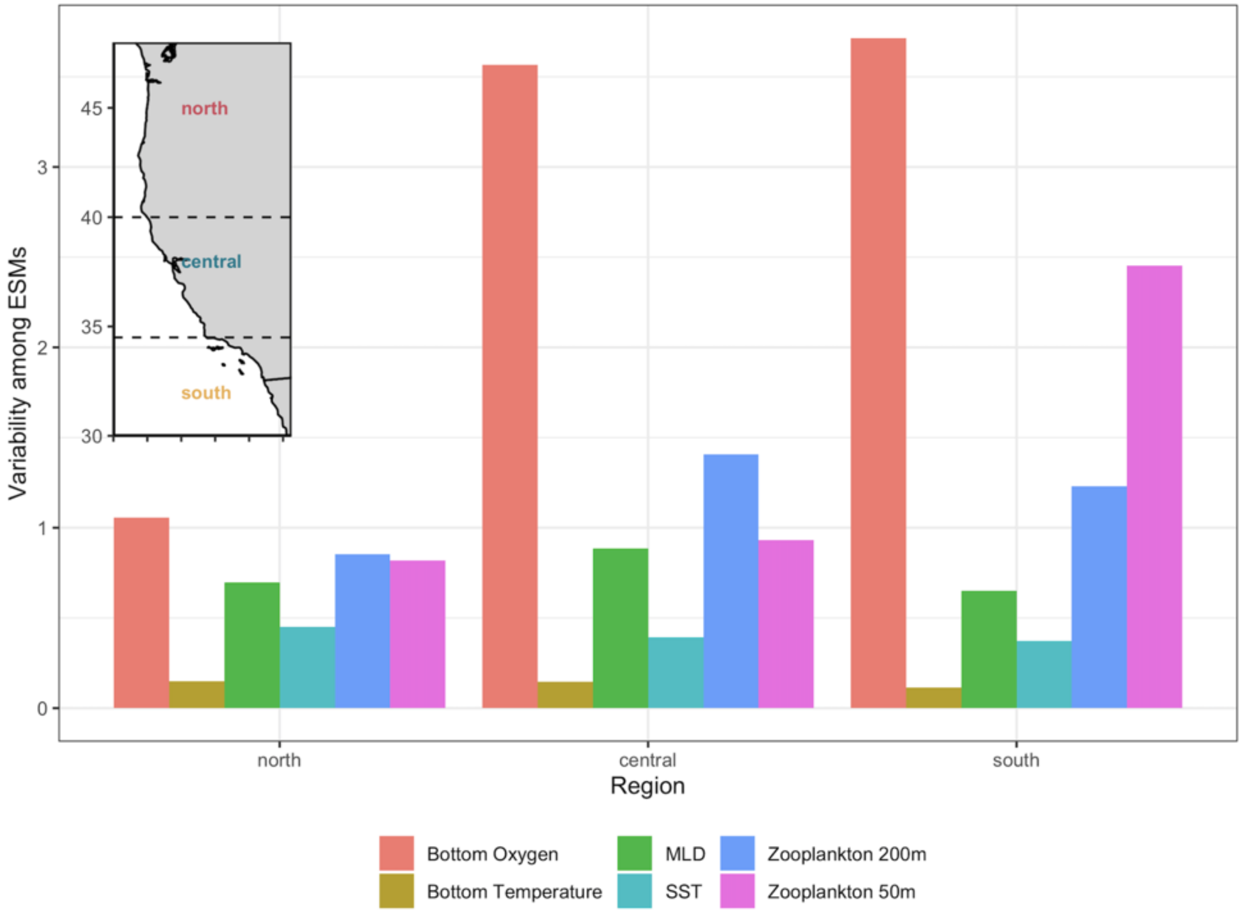

Figure S8 Variation among environmental variables for three earth system models (ESMs), shown for three regions in the California Current System. Note that variability among ESMs is lower in northern regions for most variables.
